# Multicomponent reactions (MCRs): a useful access to the synthesis of benzo-fused γ-lactams

**DOI:** 10.3762/bjoc.15.104

**Published:** 2019-05-08

**Authors:** Edorta Martínez de Marigorta, Jesús M de Los Santos, Ana M Ochoa de Retana, Javier Vicario, Francisco Palacios

**Affiliations:** 1 Departamento de Química Orgánica I, Facultad de Farmacia, University of the Basque Country, UPV/EHU Paseo de la Universidad 7, 01006, Vitoria-Gasteiz, Spain

**Keywords:** indolin-2-ones, isoindolinones, γ-lactams, multicomponent reactions, 2-oxindoles

## Abstract

Benzo-fused γ-lactam rings such as isoindolin-2-ones and 2-oxindoles are part of the structure of many pharmaceutically active molecules. They can be often synthesized by means of multicomponent approaches and recent contributions in this field are summarized in this review. Clear advantages of these methods include the efficiency in saving raw materials and working time. However, there is still a need of new catalytic systems to allow the enantioselective preparation of these heterocycles by multicomponent reactions.

## Introduction

**Figure 1:**
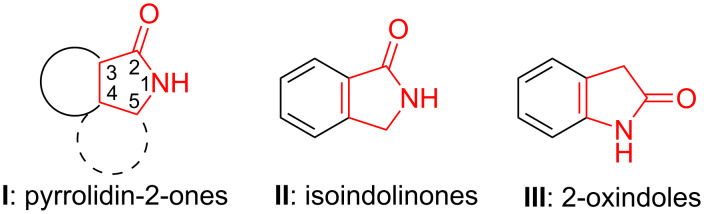
γ-Lactam-derived structures considered in this review.

Pyrrolidin-2-ones (**I**, Figure 1) are heterocycles that contain a γ-lactam ring that can be found in many biologically active compounds with natural or synthetic origin [1]. When an aryl group is fused to the 3- and 4-positions of the five-membered heterocycle, isoindolinones (**II**, Figure 1) are generated, while if such fusion takes place between the 4- and 5-positions of the γ-lactam ring, 2-oxindoles (also named as indolin-2-ones **III**, Figure 1) are formed.

**Figure 2:**
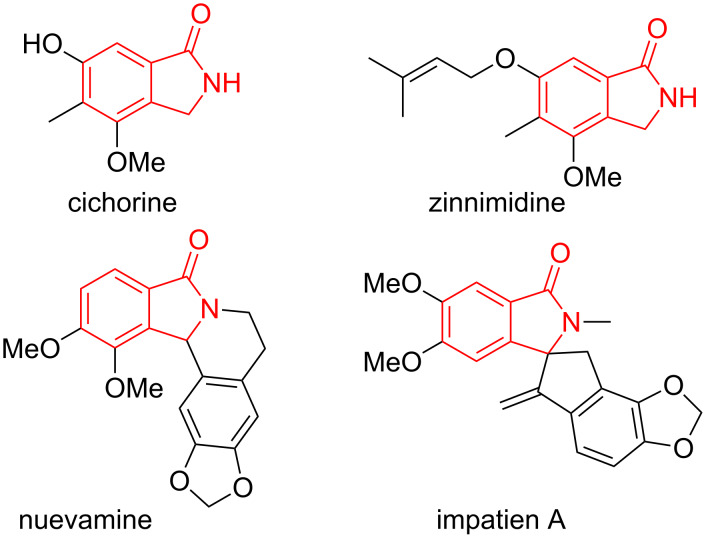
Alkaloids containing an isoindolinone moiety.

The isoindolinone structural motif is a part of the core of many natural products [2]. To cite some examples, cichorine [3] and zinnimidine [4] (Figure 2) are simple isoindolinone alkaloids, for which total syntheses have been reported [5], and nuevamine (Figure 2) is an isoindoloisoquinoline alkaloid [6–7]. Moreover, in this last decade, new compounds such as impatien A [8] (Figure 2) or daldinans B and C [9] have been discovered.

**Figure 3:**
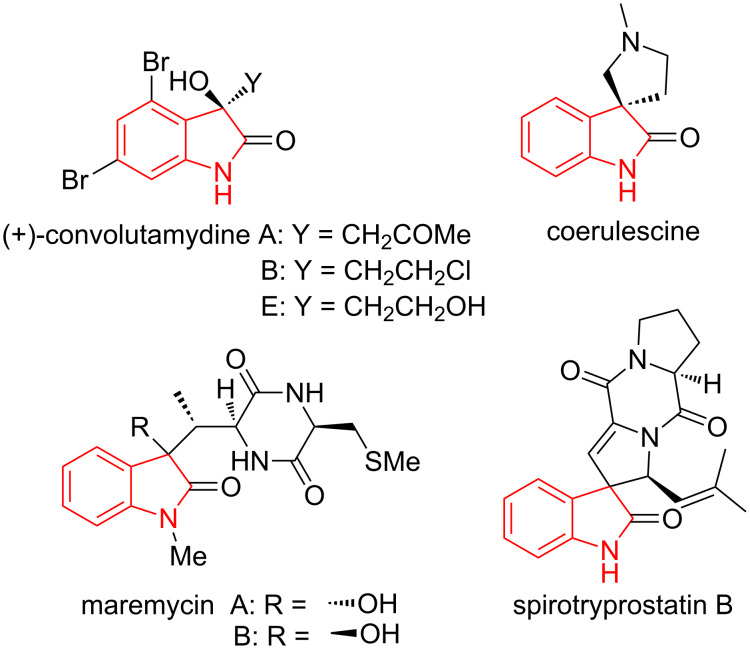
Alkaloids containing the 2-oxindole ring system.

Similarly, the 2-oxindole framework is prevalent in a wide range of natural products [10]. For example, convolutamydines [11] are alkaloids containing a dibromohydroxyoxindole moiety, isolated from the Floridian bryozoan *Amathia convulata*, while coerulescine [12] is an oxindole alkaloid isolated from *Phalaris coerulescens* (Figure 3). Likewise, maremycins [13] and spirotryprostatin B [14] have been isolated from marine *Streptomyce*s and from the fermentation broth of *Aspergillus fumigatus.*

**Figure 4:**
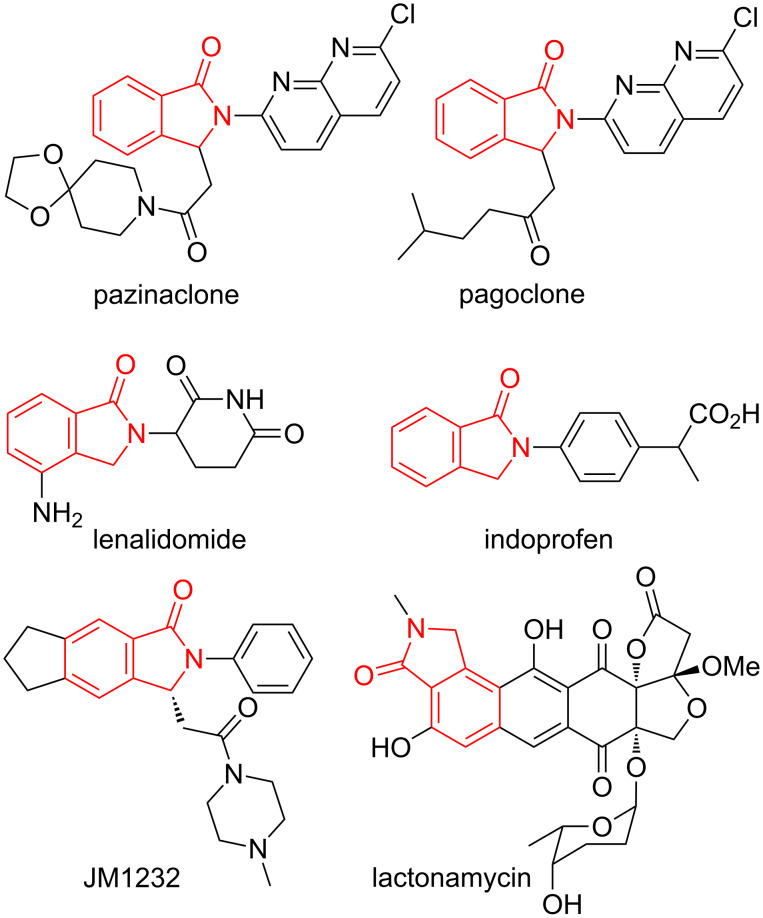
Drugs and biological active compounds containing an isoindolinone moiety.

Among benzo-fused γ-lactam chemical entities, natural and synthetic isoindolinones are a prominent class of compounds. Molecules containing the isoindolinone moiety are of pharmaceutical interest as anxiolytic (pazinaclone [15], pagoclone [16], and JM1232 [17]), anticancer (lenalidomide) [18], anti-inflammatory (indoprofen) [19] and antibiotic agents (lactonamycin) [20] (Figure 4). In addition, some isoindolinone derivatives show a large variety of biological activities [21–23], such as COX-2 inhibition [24], glucokinase activation [25], sodium channel blocking [26], antimycobacterial [27], antiproliferative [28] or carbonic anhydrase inhibition [29], as well as antifungal and antibacterial properties [30].

**Figure 5:**
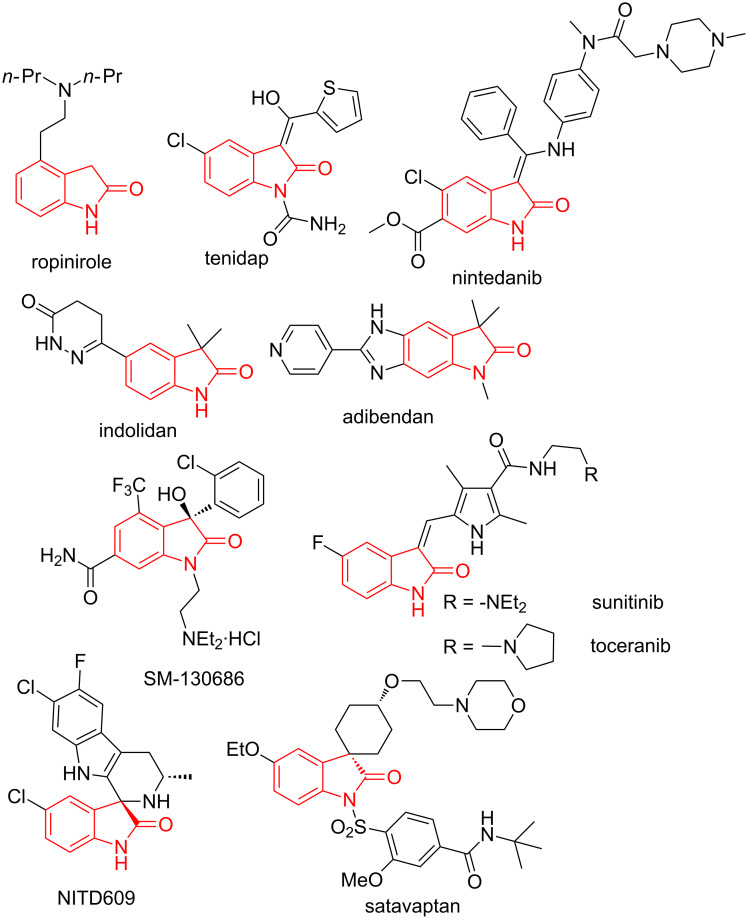
Drugs and biologically active compounds bearing a 2-oxindole skeleton.

Isomeric 2-oxindoles are another family of aromatic-fused heterocycles containing the γ-lactam unit. These molecules are often biologically active and therefore, they have found many applications in medicinal chemistry. For example, ropinirole [31] is used in the treatment of early Parkinson’s disease, nintedanib is employed against pulmonary fibrosis [32], tenidap [33] is a nonsteroidal anti-inflammatory drug (NSAID), while indolidan [34] and adibendan [35] are potent long-acting cardiotonic agents and SM-130686 is a GHSR agonist [36] (Figure 5). Other kinase inhibitors such as sunitinib [37] and toceranib [38] are also found in the market for the treatment of several tumours (Figure 5) and some others are under development or have been entered clinical trials [39]. There are also natural spirooxindole-containing complex molecules that have shown potential medicinal applications, such as NITD609, that shows antimalarial activity [40] and satavaptan, a selective V2-receptor antagonist that is useful for the treatment of cirrhosis (Figure 5) [41–42]. Additionally, methisazone is a 2-oxindole derivative that has been used as antiviral drug, especially for the prophylactic treatment of small-pox since 1965 [43] and citrinadins A and B have shown activity against murine leukaemia and human epidermoid carcinoma [44], while PF1270 A, B and C act as histamine H3 receptor ligands and, consequently, can be of therapeutic interest to treat diabetes, obesity and central nervous system disorders [45]. Besides, several hybrid molecules containing, inter alia the oxindole moiety, have been discovered and they demonstrated diverse therapeutic activities, for example, against breast [46] and colon cancer cells [47] and drug-resistant bacteria [48].

Among the many excellent recent reviews on the preparation of heterocycles by multicomponent reactions (MCRs) [49–55], the synthesis of three- and four-membered heterocycles through MCRs has been reviewed recently [56], and taking into account the increasing amount of procedures developed in the last years for the preparation of heterocycles [57–58] with interest in medicinal chemistry [59], such as those devoted to the synthesis of 5-membered γ-lactam heterocycles by means of multicomponent protocols [60], we believe that this field also deserves an in-depth revision that would benefit the researchers working in the synthesis of heterocycles. Especially interesting are the applications of these MCRs [61–64] in combinatorial chemistry [65] and diversity-oriented synthesis [66], where structurally diverse compound libraries can be rapidly synthesized.

Therefore, this article reviews the procedures disclosed recently for the multicomponent synthesis of isoindolinones **II** and 2-oxindoles **III** (Figure 1), where the setup of the γ-lactam core takes place during the key process.

MCRs involve the simultaneous reaction between three or more reactants to deliver products that include significant fragments of all the substrates in their structure. Those starting materials must be added all together in the reaction container and, therefore, other approaches [67–69] featuring sequential (domino, tandem or cascade) [70–71] reactions, where one intermediate is initially preformed before additional reagents are added, are not included in this review.

## Review

### Isoindolinones

An extensive range of synthetic methods have been developed for the preparation of isoindolinone derivatives [72–75], although only a few make use of multicomponent approaches.

Most of the multicomponent syntheses of this type of heterocycles employ a benzoic acid derivative as one of the substrates of the reaction, together with an amine and a third reagent that provides the carbon atom needed to complete the cyclic moiety.

**Scheme 1:**
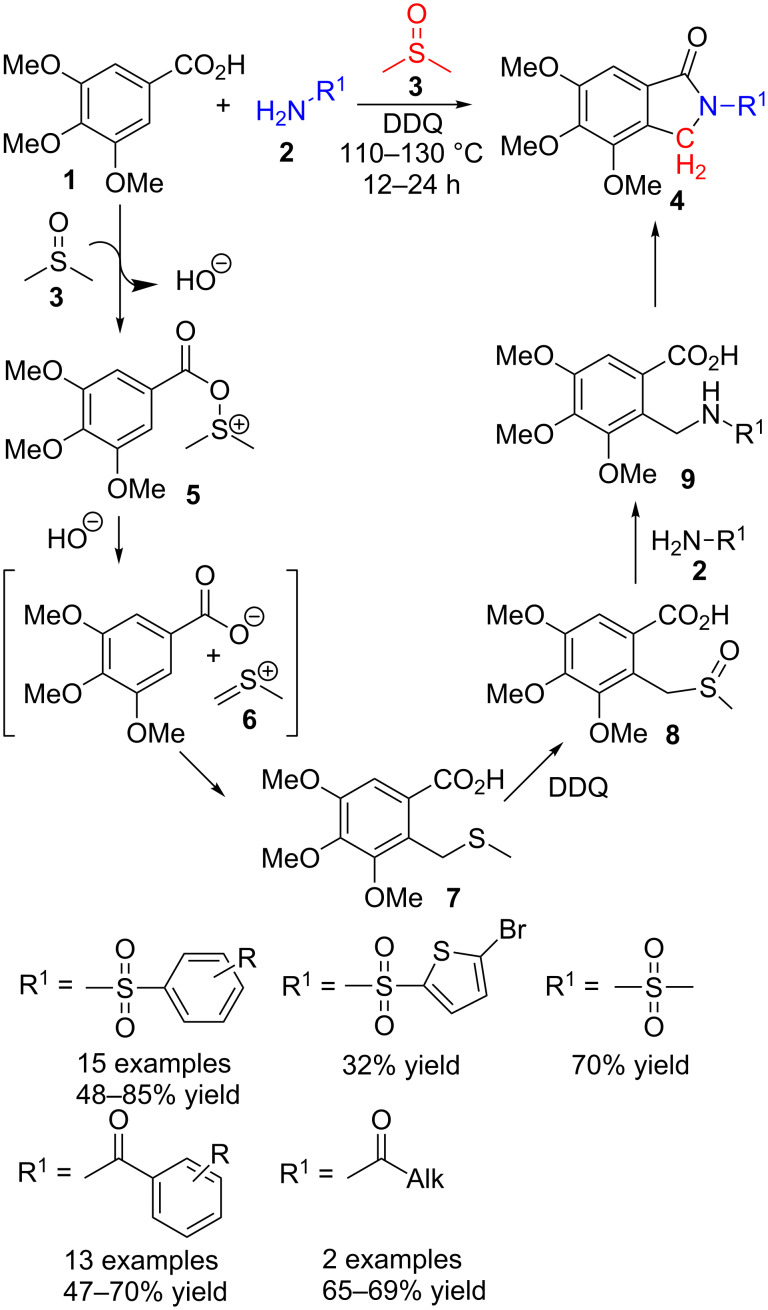
Three-component reaction of benzoic acid **1**, amides **2** and DMSO (**3**).

Thus, Shi et al. [76] reacted benzoic acid derivatives **1**, amides **2** and DMSO (**3**) in the presence of DDQ as oxidant and without any metal catalyst. Through a tandem three-component cross-dehydrogenative coupling (CDC), they prepared, in a single step, more than thirty isoindolinone derivatives **4**, including those originated from sulfonamides and carboxamides (Scheme 1). The scope of the reaction includes aromatic, some aliphatic and one heteroaromatic derivative with yields ranging from moderate to good. It is remarkable that no catalysts are needed in this transformation. Unfortunately, other aromatic acids bearing substituents different from three methoxy groups did not produce any detectable amounts of isoindolinone.

Based on several additional experiments, the authors propose a tentative mechanism based on a Pummerer-type rearrangement. First, dimethyl sulfoxide (**3**) and carboxylic acid **1** combine to render ester **5** that decomposes, giving a thionium derivative of DMSO (**6**, Scheme 1). This electrophilic derivative would react with the nucleophilic aromatic ring in a Friedel–Crafts alkylation process, thus incorporating the carbon atom in a formal C(sp^2^)–H/C(sp^3^)–H cross-dehydrogenative coupling. Finally, an oxidation of sulfide **7** to sulfoxide **8** and the subsequent attack of amide **2** with cleavage of the C–S bond and formation of **9**, followed by an intramolecular cyclic amide formation, would produce isoindolinone derivatives **4**.

**Scheme 2:**
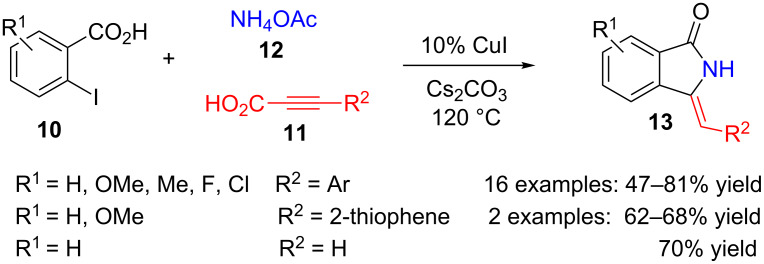
Copper-catalysed three-component reaction of 2-iodobenzoic acids **10**, alkynylcarboxylic acids **11** and ammonium acetate (**12**).

Employing benzoic acids **10** *ortho*-substituted with iodine along with alkynylcarboxylic acids **11** and ammonium acetate (**12**), in the presence of caesium carbonate and copper iodide as catalyst (10%), a series of 3-methyleneisoindolin-1-ones **13** were obtained (Scheme 2) [77]. Several aromatic and heteroaromatic substituents can be introduced in the exocyclic methylene position with high regioselectivity. It is noteworthy that arylalkynylcarboxilic acids **11** (R^2^ = Ar) can be obtained easily by the coupling reaction of propiolic acid and aryl halides [78].

Furthermore, the authors have been able to carry out the reaction in a sequential manner starting from propiolic acid and aryl iodides in the presence of caesium carbonate and a palladium catalyst. Next, addition of 2-iodobenzoic acid and ammonium acetate leads to the formation of isoindolinone derivative **13** in 55–65% global yields, without isolating any intermediate.

**Scheme 3:**
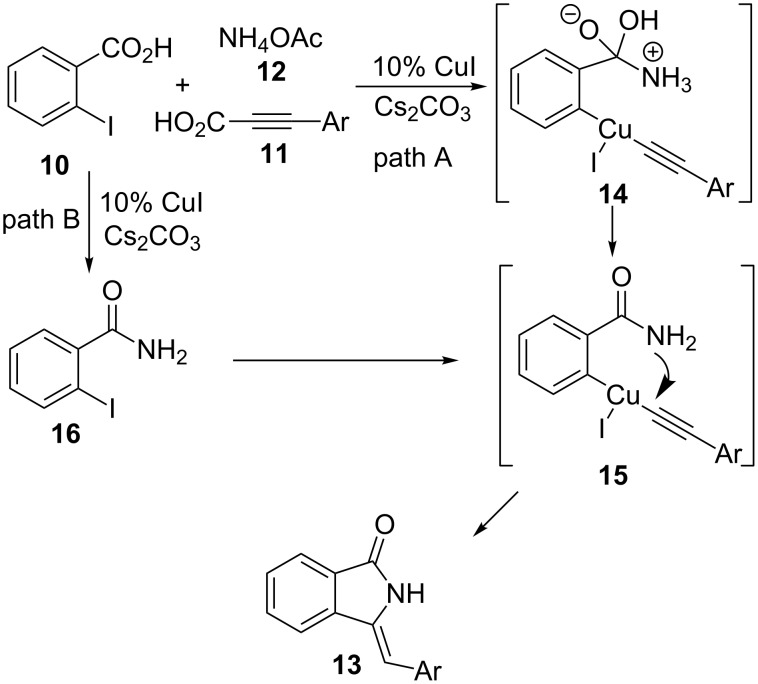
Proposed mechanism for the formation of methylene isoindolinones **13**.

Regarding the reaction mechanism, the authors have ruled out that phthalide must be an intermediate of the reaction and they proposed the two pathways illustrated in Scheme 3. In the first one (path A), decarboxylative coupling to form intermediates **14** and **15**, followed by a cyclization take place, while in the second path (B), the first step seems to be the formation of amide **16**.

**Scheme 4:**
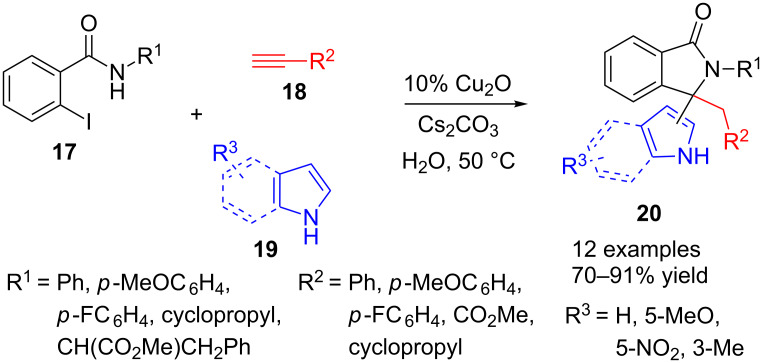
Copper-catalysed three-component reaction of 2-iodobenzamide **17**, terminal alkyne **18** and pyrrole or indole derivatives **19**.

Indeed, another multicomponent approach to isoindolinones uses iodobenzamides **17** as starting materials (Scheme 4). In this case, copper catalyst and alkynes are also used but, unlike the above method, alkynes **18** are monosubstituted. The third component is an indole or pyrrole derivative **19** and the result of the reaction is a 3,3-disubstituted isoindolinone derivative **20**, which contains a newly formed quaternary centre [79].

The reaction takes places in water, even in the presence of air, and with nanodomain cuprous oxide as an inexpensive and reusable catalyst. The scope includes aryl, alkyl and carboxyl groups at 2- and 3-positions of the lactam ring. Pyrrole reacts across the 2-carbon while indole derivatives do it through the carbon at 3-position. Although a quaternary stereocentre is created in the process, no attempts to make this synthesis in a stereoselective fashion were reported.

A tentative mechanism is proposed, based in a Sonogashira coupling of iodobenzamide **17** and copper acetylide, in a similar way to that described in Scheme 3. In this case, the coupled alkyne moiety is again activated by Cu(I) and then base-promoted cyclization occurs. A new copper complex formation with the alkene analogue to **13** (see Scheme 3) facilitates the aromatic nucleophilic substitution by indole or pyrrole, leading to final lactams **20**.

**Scheme 5:**
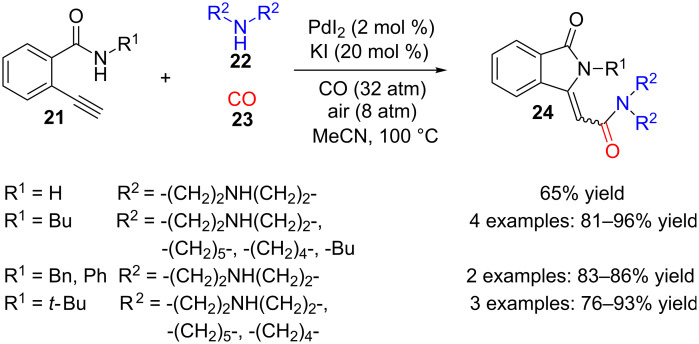
Palladium-catalysed three-component reaction of ethynylbenzamides **21**, secondary amines **22** and CO (**23**).

This mechanism is partially corroborated by the following multicomponent synthesis where benzamide **21**, *ortho*-functionalized with a terminal alkyne group (Scheme 5), a secondary amine **22** and carbon monoxide (**23**) react to produce 3-methyleneisoindolinones **24** [80]. A palladium catalyst in acetonitrile, and a high pressure of both, CO and air, are needed in order to perform an oxidative carbonylation on 2-ethynylbenzamide **21**.

Up to ten compounds were obtained with yields ranging from 65 to 96%. Butyl, phenyl, benzyl or absence of substitution on the amide nitrogen produced, in most of the cases, the *Z*-isomer as the sole or main product, while *tert*-butyl derivatives gave the *E*-isomer selectively. On the secondary amine side, morpholine, piperidine, pyrrolidine and dibutylamine all rendered the reaction efficiently.

**Scheme 6:**
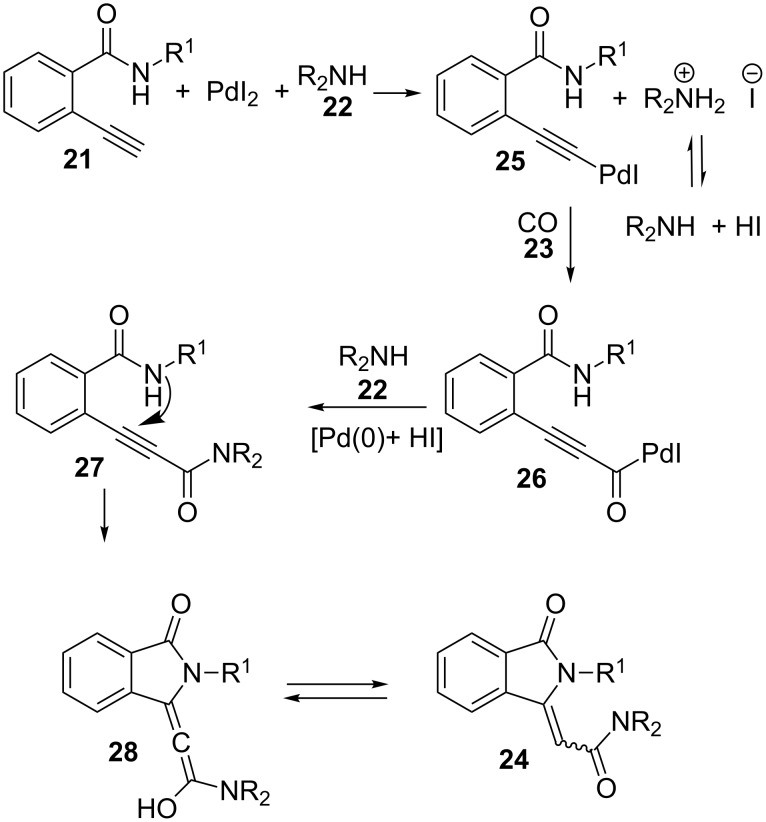
Proposed mechanism for the formation of methyleneisoindolinones **24**.

A plausible explanation for the mechanism of this transformation implies initial formation of an alkynylpalladium intermediate **25** followed by carbonylation to render acylpalladium **26** (Scheme 6). Reductive substitution of palladium by amine **22** furnishes diamide derivative **27** and Pd(0), which is reoxidized to Pd(II) again by HI and oxygen. Then, intramolecular conjugate addition of benzamide nitrogen onto the 2-ynamide generates the final cyclization product **24** through allene intermediate **28**. Taking into account that the reaction does not take place with internal alkynes, the authors conclude that a terminal alkyne is necessary for the formation of the first alkynylpalladium complex **25** in the proposed pathway.

**Scheme 7:**
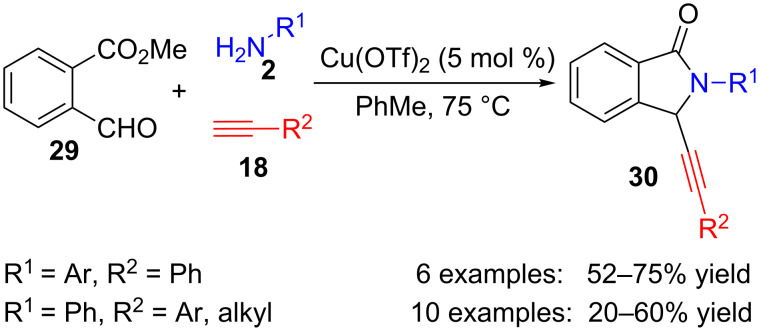
Copper-catalysed three-component reaction of formyl benzoate **29**, amines **2** and alkynes **18**.

Other benzoic acid derivatives such as those bearing a formyl substituent at the *ortho* position also take part in several multicomponent cyclizations leading to isoindolinones. Thus, 2-formylbenzoate **29**, primary amines **2** and terminal alkynes **18** react under copper catalysis to furnish several propargylisoindolinones **30** with modest to good yields (Scheme 7) [81]. Although aryl- and alkylacetylenes can be used in this method, only aromatic amines **2** which are not *ortho*-substituted work well under these reaction conditions.

The reaction probably takes place through an addition of copper acetylide, generated from terminal alkyne and copper, to the imine formed by the reaction between the amine and the formyl group. Then, the secondary propargylamine intermediate would act as a nucleophile in a cyclization process to form the lactam ring.

**Scheme 8:**
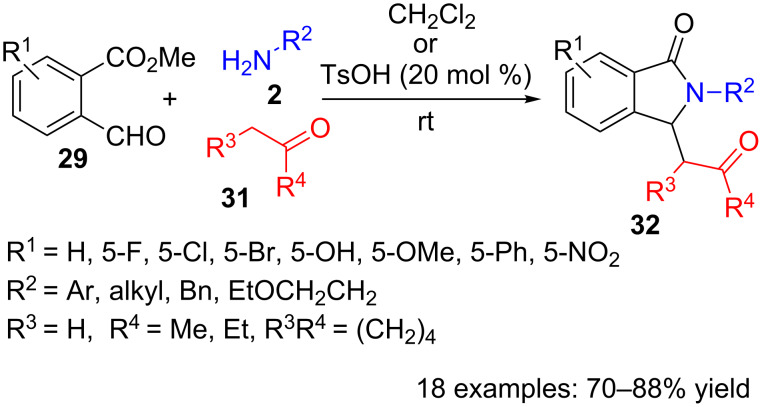
Copper-catalysed three-component reaction of formylbenzoate **29**, amines **2** and ketones **31**.

The same formylbenzoate **29** has also been used in another three-component synthesis along with amines **2** and ketones **31** (Scheme 8) [82]. This Mannich/lactamization reaction achieves good yields for a broad scope of 3-substituted isoindolinones **32**, in either catalyst-free conditions or using *p*-toluenesulfonic acid. *Ortho*- and *meta*-substituted anilines **2** did not produce isoindolinones **32**, and aliphatic amines only reacted when *p*-toluenesulfonic acid was present. The reaction has also been applied to 1,3-dicarbonyl compounds, however, only residual amounts of isoindolinones **32** were detected and deamination products became predominant.

Similarly to the above mentioned method, the first step in this approach is probably the imine formation by reaction of amine **2** with the aldehyde functionality of benzoate **29**. Next, addition of the enol form of ketone **31** onto the imine would provide a Mannich intermediate amine, which can intramolecularly attack the ester function, giving rise to lactam **32**.

As pointed out above, unfortunately this methodology did not work properly when it was first applied to 1,3-dicarbonyl compounds. Nevertheless, more recently, two research groups have disclosed, nearly simultaneously, the three-component cyclization of 2-formylbenzoic acids, primary amines and a 1,3-dicarbonyl compounds.

**Scheme 9:**
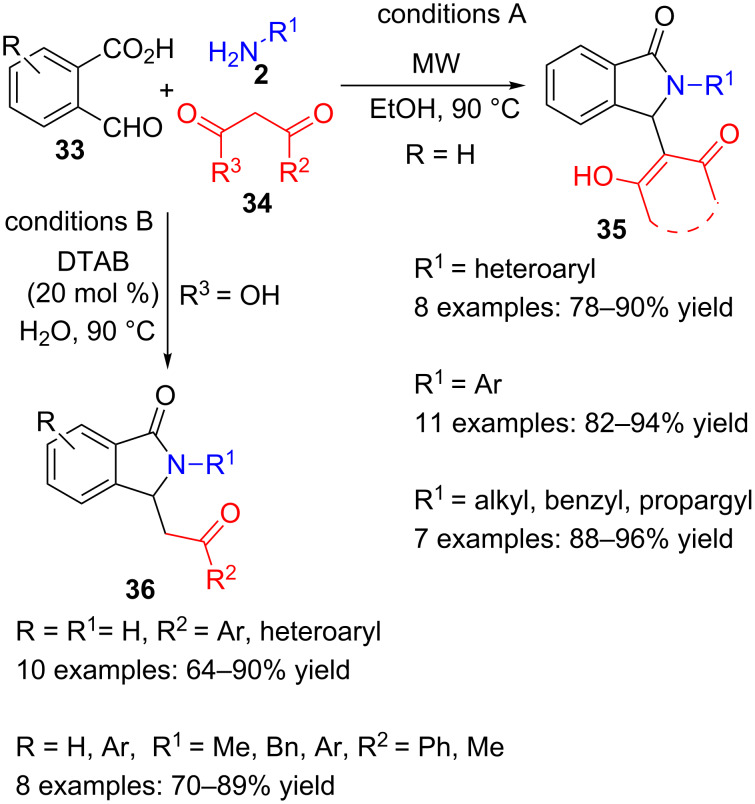
Non-catalysed (A) and phase-transfer catalysed (B) three-component reactions of formylbenzoic acids **33**, amines **2** and 1,3-dicarbonyl derivatives **34**.

The first proposal uses 2-formylbenzoic acid **33** (R = H, conditions A, Scheme 9), cyclic aliphatic and aromatic diketones **34** such as dimedone (R^2^R^3^ = -CH_2_CMe_2_CH_2_-) as the 1,3-dicarbonyl partner and a variety of aromatic, heteroaromatic and aliphatic amines in ethanol, under microwave heating, and without metal catalyst [83]. Very good yields of **35** are obtained in a very simple and cost-effective manner.

A rather similar approach was disclosed shortly later by Han and co-workers [84], who used β-ketoacids (**34** R^3^ = OH, conditions B, Scheme 9) instead of diketones and a quaternary ammonium salt as catalyst in water. In this multicomponent decarboxylative alkylation/cyclization process, they prepared several lactam derivatives **36** with good yields.

**Scheme 10:**
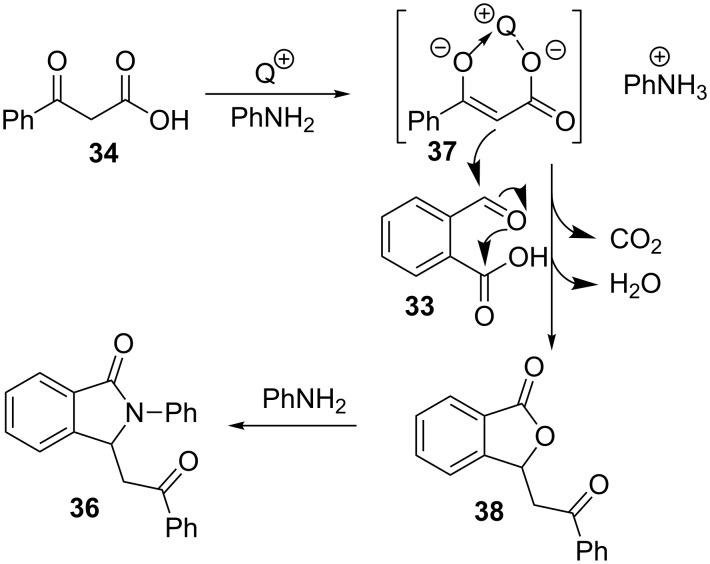
Proposed mechanism for the formation of isoindolinones **36**.

While the first research group suggests that the reaction would start with the formation of an imine intermediate between the aldehyde function of **33** and the amine **2**, followed by a nucleophilic attack by the diketone and a final intramolecular cyclization, the Han group proposes a different pathway, with an initial deprotonation of ketoacid **34** (R^2^ = Me, Ar, R^3^ = OH) and a subsequent addition of enolate **37** onto the aldehyde moiety in **33**, with concomitant decarboxylation and cyclization to form a phthalide intermediate **38** (Scheme 10). Then, the known amine-substitution reaction would transform phthalide **38** into isoindolinone **36**.

A conceptually very similar procedure has been described by Singh and co-workers [85], who used formylbenzoate **29** and preformed enol ethers instead of ketones in a Mukaiyama–Mannich lactamization reaction catalysed by zinc or copper under mild conditions. A large amount of diverse isoindolinones **32** (thirty-four examples) can be built in this manner, although, once again, *ortho*-substituted anilines **2** did not render the cyclic product, as the final lactamization step is probably impeded by sterical reasons. On the other hand, silyl enol ethers of acetone, acetophenone, methyl acetate, 2-hydroxyfuran and cyclohexanone worked well, providing isoindolinone **32** with yields ranging from 64 to 85%.

**Scheme 11:**
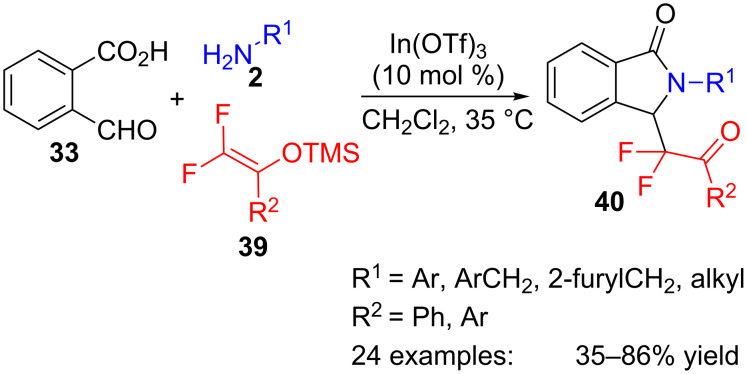
Three-component reaction of formylbenzoic acid **33**, amines **2** and fluorinated silyl ethers **39**.

A variation of the above approach that makes use of fluorinated silyl ethers **39**, has been applied to the synthesis of analogous fluorinated isoindolinones **40** (Scheme 11) [86]. In this case, formylbenzoic acid **33**, a variety of aromatic, aliphatic and heteroaromatic amines **2** and trimethylsilyl enol ethers **39** are combined in a three-component Mannich/lactamization reaction in the presence of an indium catalyst to yield twenty four 3-difluoroalkylisoindolinone derivatives **40**.

**Scheme 12:**
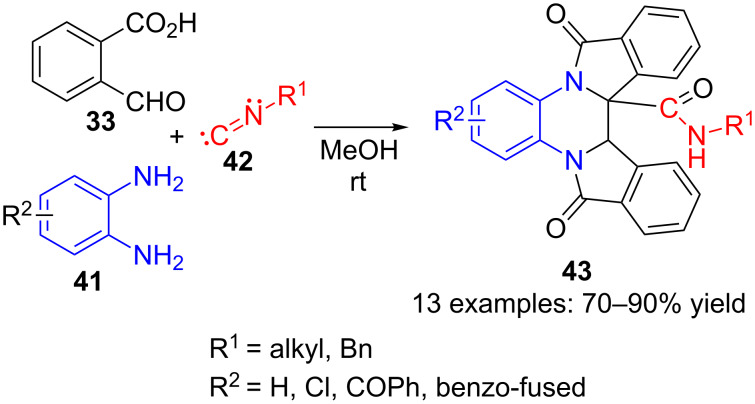
Three-component Ugi reaction of 2-formylbenzoic acid (**33**), diamines **41** and isocyanides **42**.

The starting 2-formylbenzoic acid **33** has been also employed in Ugi-type multicomponent reactions with amines and isocyanides by several research groups to make highly functionalized lactams. After the pioneering works by Ley [87] and Zhang [88], other contributions have been reported in the last years. For example, Shaabani et al. [89] used diamines **41**, isocyanides **42** and two equivalents of 2-formylbenzoic acid (**33**) in an Ugi three-component reaction with methanol as solvent at room temperature to afford tetrahydrodiisoindoloquinoxalinecarboxamides **43** (Scheme 12).

**Scheme 13:**
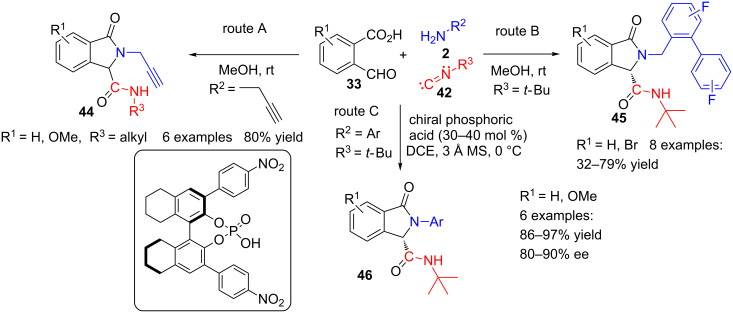
Non-catalysed (A, B) and chiral phosphoric acid promoted (C) three-component Ugi reactions of formylbenzoic acids **33**, amines **2** and isocyanides **42**.

More recently, Shafiee et al. [90] utilized propargylamine as the cycle-nitrogen delivering component and then submitted the obtained isoindolinones **44** (route A, Scheme 13) to an additional cyclization to furnish a pyrazinoisoindoledione derivative. Kajanus and co-workers [91] prepared eight isoindolinone derivatives **45** by this method with yields ranging from 32 to 79% (route B, Scheme 13).

They also made several analogues of these compounds by a sequential Ugi/Diels–Alder approach and, in this context, they were able to separate the enantiomers using chiral chromatography. Some of these compounds showed good in vitro potency blocking the cardiac ion channel Kv1.5 and, therefore, are promising agents to treat atrial fibrillation. Nevertheless, the most outstanding contribution is the first enantioselective Ugi synthesis of isoindolinones **46** catalysed by a chiral phosphoric acid, reported by D.-X. Wang, M.-X. Wang, J. Zhu and co-workers (route C, Scheme 13) [92]. They obtained very good yields and remarkable enantiomeric excesses, which result, according to the authors, from a dynamic kinetic resolution of the initially formed Ugi adduct.

**Scheme 14:**
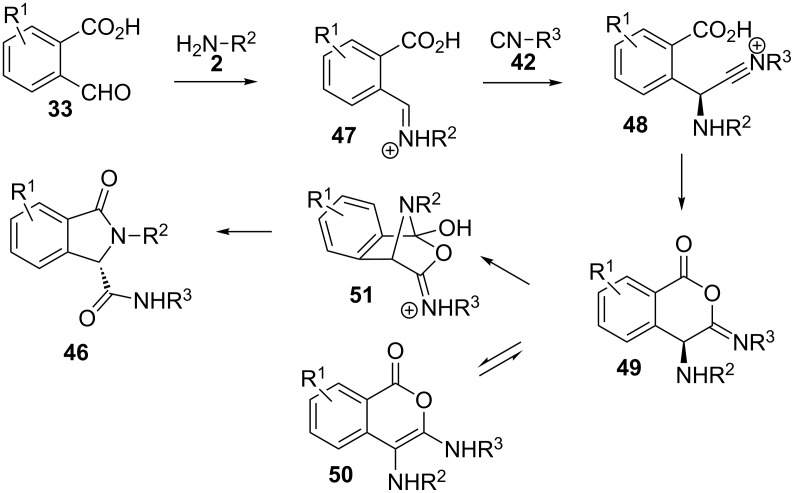
Proposed mechanism for the enantioselective formation of isoindolinones **46**.

Indeed, the plausible mechanism for the reaction implies the condensation of amine **2** and aldehyde **33** to form iminium salt intermediate **47** (Scheme 14). Next, addition of isocyanate **42** would supply the corresponding nitrilium intermediate **48**, which then can be trapped intramolecularly by the carboxylate moiety, thus furnishing isocumarine **49**. According to several control experiments performed by the authors, the imine **49**–enamine **50** tautomerization seems to happen faster than the Mumm rearrangement that would lead to isoindolinone **46** through the bridged intermediate **51**. Therefore, this mechanistic path shows that the enantioselectivity of the reaction is a consequence of a dynamic kinetic resolution of enamine **50**.

**Scheme 15:**
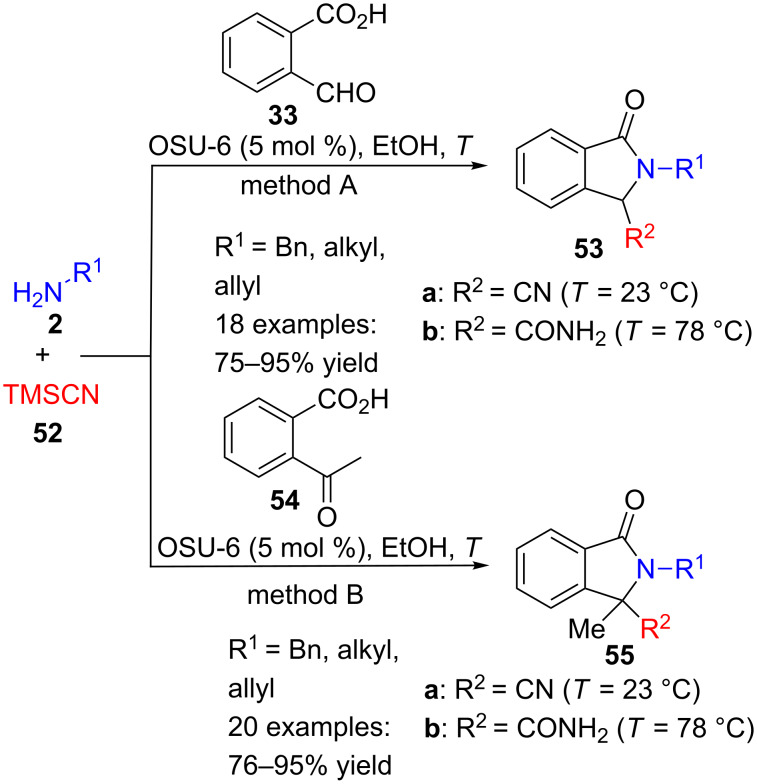
Three-component reaction of benzoic acids **33** or **54**, amines **2** and TMSCN (**52**).

Cyanide can also be used, instead of isocyanide, in an analogous three-component reaction, to afford isoindolinones **53** substituted with nitrile or carboxamide groups (Scheme 15, method A) [93]. Trimethylsilylcyanide (**52**), and benzyl-, alkyl- and allylamines **2** were reacted with 2-formylbenzoic acid (**33**) in the presence of OSU-6, a mesoporous silica performing as a green Lewis acid catalyst for this transformation. At room temperature, the product of this environmentally friendly Strecker reaction is nitrile derivative **53** (R^2^ = CN, Scheme 15, method A), while at reflux carboxamide **53** (R^2^ = CONH_2_, Scheme 15, method A) is obtained. Notoriously, aromatic amines **2** did not work under these conditions and, in place of isoindolinones **53**, isobenzofuranones were isolated. The method was extended to the corresponding acetophenone derivative **54** and, in this case, quaternary nitriles and carboxamides **55** were prepared in good yields (Scheme 15, method B).

This Strecker approach has also been performed by using scandium catalyst (Sc(OTf)_3_, 2.5 mol %) and starting from ester **29**. Under these conditions, not only benzyl or alkyl, but also a wide range or *ortho*-, *meta*- and *para*-arylamines **2** can be used at room temperature to make 3-oxoisoindolinone-1-carbonitriles **53a** in very good yields (22 examples, 82–97% yields) [94].

On the contrary, another Strecker multicomponent synthesis, between 2-formylbenzoate **29**, benzylamine and potassium cyanide, carried out under mechanochemical conditions and zinc catalysis, only produced a 31% yield of the corresponding cyanoisoindolinone **53a** (R^1^ = Bn), along with 34% yield of benzyl phthalimide, probably formed by air oxidation of **53a** [95].

**Scheme 16:**
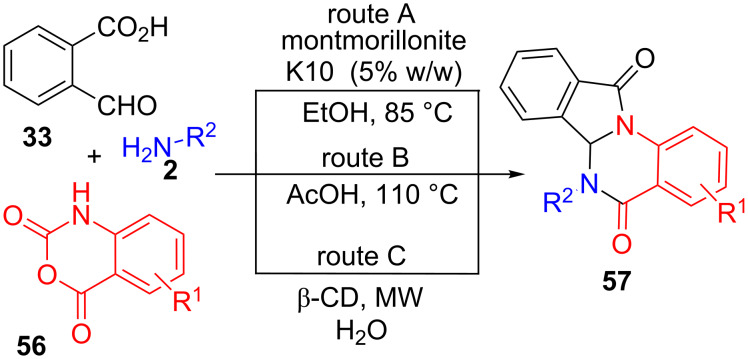
Several variations of the three-component reaction of formylbenzoic acids **33**, amines **2** and isatoic anhydrides **56**.

2-Formylbenzoic acid (**33**) has also been used in another type of three-component cyclization, along with amines **2** and isatoic anhydrides **56**, leading to isoindoloquinazolinone derivatives **57**, a kind of heterocycle containing five- and six-membered fused *N*-heterocyclic rings (Scheme 16), including the γ-lactam unit. The first synthesis of this class of compounds was reported by Pal et al. [96] with the aid of montmorillonite K10 as a recyclable catalyst in ethanol. With these environmentally friendly conditions, they reported the preparation of twelve analogues of **57** with good yields (72–95%, R^1^ = H, R^2^ = alkyl, benzyl, aryl) (route A, Scheme 16). Some of these molecules were able to inhibit tumour necrosis factor-alpha (TNF-α) in vitro, therefore, showing potential medicinal applications.

A similar synthetic approach was applied by another research group, using acetic acid instead of ethanol as a solvent and without any other catalyst (route B, Scheme 16) [97]. By this way, seventeen compounds of type **57** were obtained with yields ranging from 80 to 92% (R^1^ = H, R^2^ = H, alkyl, benzyl, aryl). Another improvement on this multicomponent approach, making use of β-cyclodextrine as promoter, water as a solvent, and microwave heating (route C, Scheme 16) [98]. Under these neutral conditions, they prepared up to nineteen compounds (60–95%, R^1^ = H, Cl, R^2^ = alkyl, benzyl, aryl), including two derivatives containing chlorine atoms in the portion coming from isatoic anhydride **56**.

**Scheme 17:**
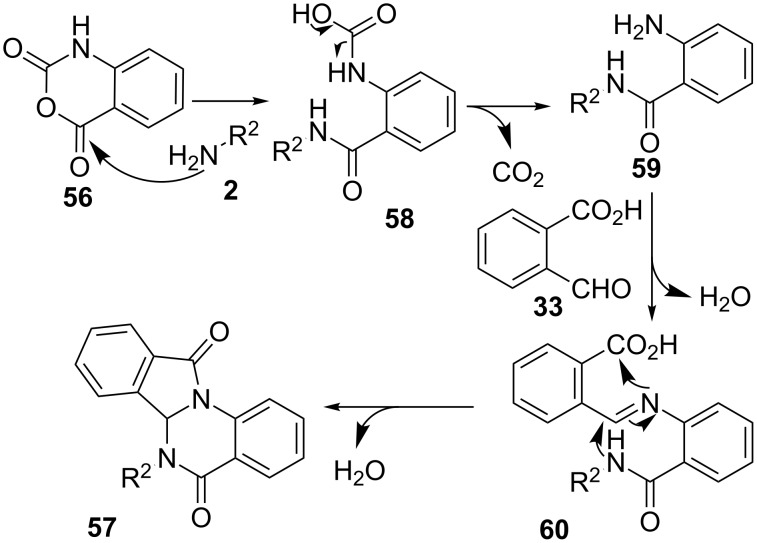
Proposed mechanism for the synthesis of isoindoloquinazolinones **57**.

In all cases, the most likely mechanism is initiated by a nucleophilic attack of amine **2** onto the anhydride carbonyl with opening of the cycle to form carbamic acid derivative **58** and loss of CO_2_ to give the intermediate 2-aminobenzamide **59** (Scheme 17). Condensation with the aldehyde function in **33**, would originate imine intermediate **60**, which then takes part in an intramolecular double cyclization to furnish the final heterocyclic substrate **57**.

**Scheme 18:**
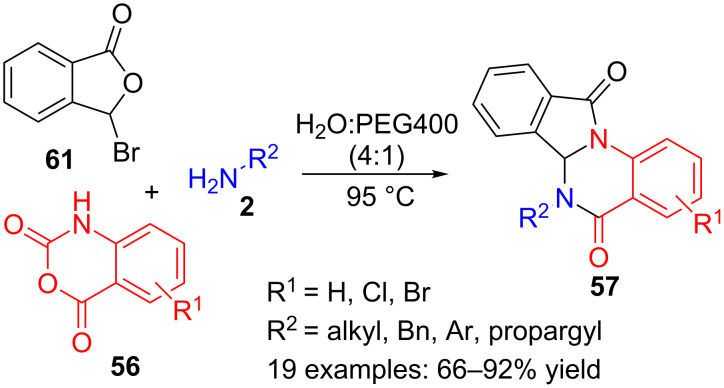
Three-component reaction of isobenzofuranone **61**, amines **2** and isatoic anhydrides **56**.

A variation of the previous procedure has been disclosed, where 2-formylbenzoic acid (**33**) is replaced by bromoisobenzofuranone **61**, also leading to isoindoloquinazoline derivatives **57** (Scheme 18) [99]. After optimization of the reaction conditions, a 4:1 combination of water and PEG-400 was chosen as the best solvent and an array of nineteen isoindoloquinazolinones **57** with yields ranging from 66 to 92% was prepared. The mechanism is presumably very similar to that of the above reaction (Scheme 17), where isobenzofuranone **61** plays the role of formylbenzoic acid **33**.

**Scheme 19:**
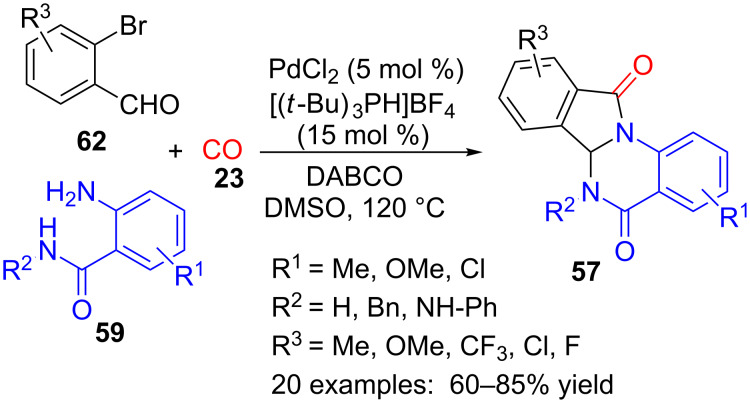
Palladium-catalysed three-component reaction of 2-aminobenzamides **59**, 2-bromobenzaldehydes **62** and CO (**23**).

Another multicomponent approach to isoindoloquinazolinones **57** is the palladium-catalysed reaction of 2-aminoamides **59**, 2-bromobenzaldehydes **62** and carbon monoxide (**23**) at atmospheric pressure, with the assistance of DABCO as base and tri(*tert*-butyl)phosphonium tetrafluoroborate as ligand (Scheme 19) [100].

A variety of substituents in the benzene rings (R^1^, R^3^) are compatible with the reaction conditions, but heteroaromatic analogues of aldehyde **62**, such as 2-bromonicotinaldehyde or 2-bromothiophene-3-carbaldehyde did not produce the desired product. On the other hand, 2-aminoquinoline-3-carboxamide also reacted under these conditions to produce the corresponding isoindoloquinazolinone analogue of **57**.

**Scheme 20:**
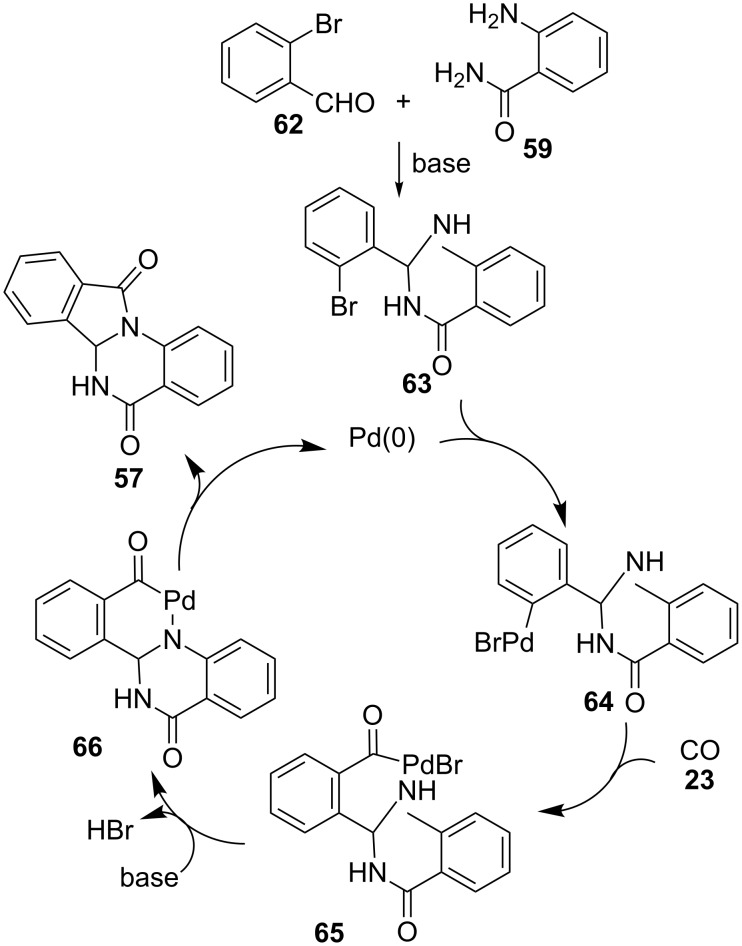
Proposed mechanism for the palladium-catalysed synthesis of isoindoloquinazolinones **57**.

Some control experiments allowed the authors to propose the mechanism as follows (Scheme 20). First, cyclocondensation of 2-aminobenzamide (**59**) with 2-bromobenzaldehyde (**62**) to form intermediate **63** is followed by oxidative addition of Pd(0) to provide palladium complex **64**. Then, insertion of CO (**23**) in the C–Pd bond furnishes an acylpalladium complex **65**, which, after elimination of hydrogen bromide and subsequent reductive elimination of palladium from intermediate **66**, affords **57** with regeneration of Pd(0).

**Scheme 21:**
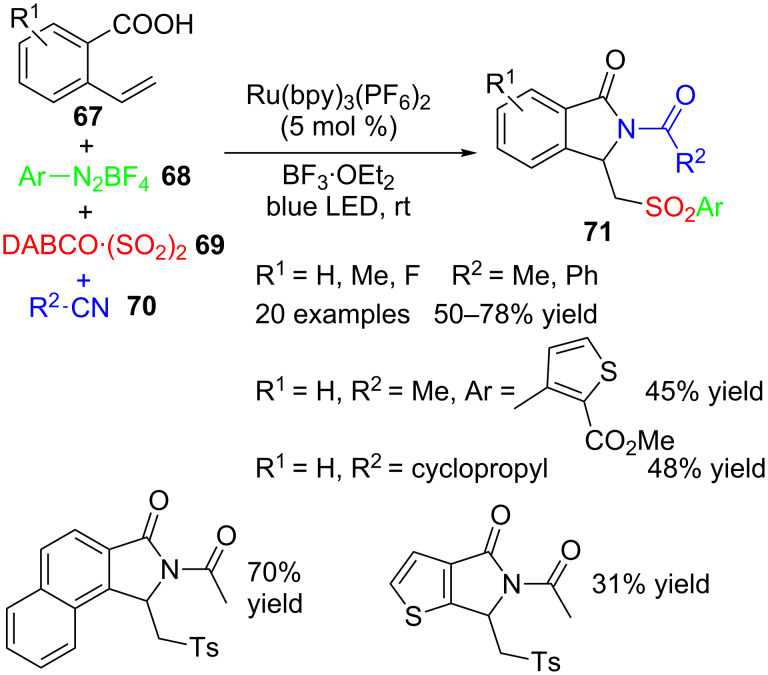
Four-component reaction of 2-vinylbenzoic acids **67**, aryldioazonium tetrafluoroborates **68**, DABCO·(SO_2_)_2_ (**69**) and nitriles **70**.

2-Vinylbenzoic acids **67** are also appropriate substrates for the preparation of isoindolinones **71** through a four-component reaction with aryldiazonium tetrafluoroborates **68**, DABCO·(SO_2_)_2_ (**69**) and nitriles **70** under Ru(IV) photocatalysis with visible light and in the presence of a Lewis acid (Scheme 21) [101].

**Scheme 22:**
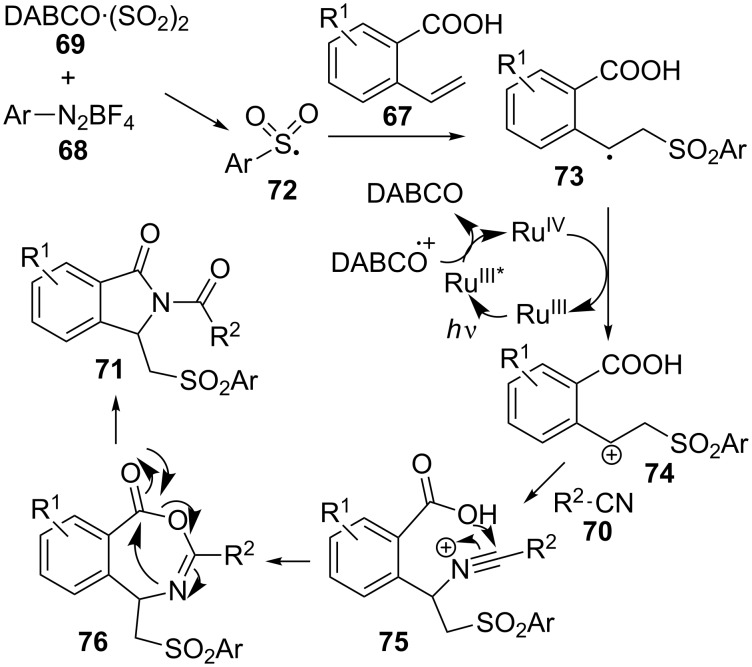
Plausible mechanism for the formation of isoindolinones **71**.

Up to 24 isoindolinone derivatives were obtained, bearing a wide variety of aryl moieties at the sulfonyl group, including a thiophene derivative. With the aid of several dedicated experiments, the researchers proposed a mechanism initiated by the formation of an arylsulfonyl radical **72**, which then would add to the alkene moiety in **67** to produce a radical intermediate **73** (Scheme 22). The photocatalyst-assisted oxidation of this radical would give rise to the corresponding cation **74**, which would add to the nucleophilic nitrile **70**. Intramolecular nucleophilic attack of the carboxy group in **75** followed by rearrangement of intermediate **76** delivered isoindolinone derivatives **71**.

**Scheme 23:**
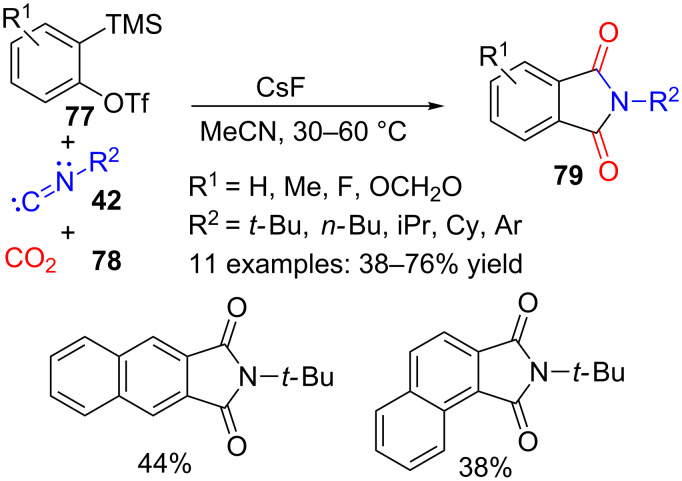
Three-component reaction of trimethylsilylaryltriflates **77**, isocyanides **42** and CO_2_ (**78**).

*Ortho*-functionalized benzoic acids have also been prepared in situ and used for a three-component transition-metal-free synthesis of phthalimides **79** induced by fluoride (Scheme 23) [102]. The reaction makes use of 2-(trimethylsilyl)aryl triflates **77**, isocyanides **42** and CO_2_ (**78**), and takes place in acetonitrile as solvent and without the need of any transition metal. Different symmetrically and unsymmetrically substituted aryne precursors **77** and alkyl and aryl isocyanides **42** produced thirteen benzamide derivatives **79**, with moderate to good yields.

**Scheme 24:**
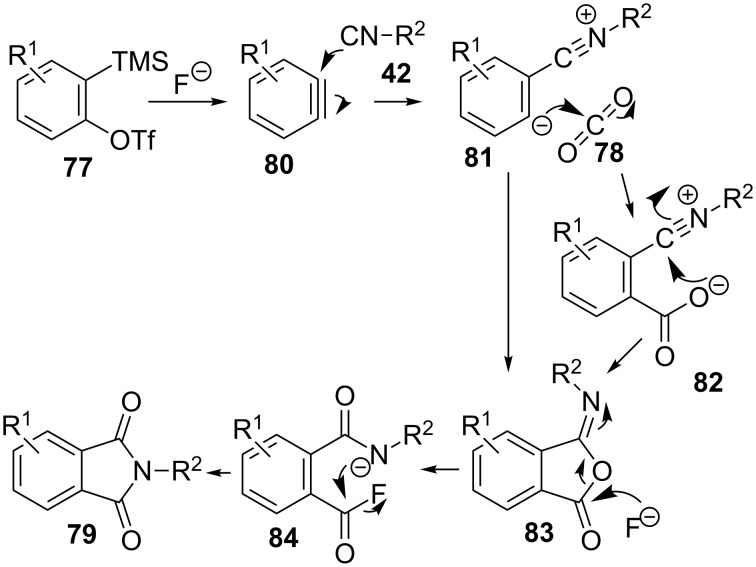
Plausible mechanism for the three-component synthesis of phthalimides **79**.

The plausible mechanism for this transformation (Scheme 24) would start with the formation of the reactive aryne **80** which is first trapped with the nucleophilic isocyanide to form **81** and then with the electrophilic CO_2_ (**78**), to furnish an intermediate benzoic acid derivative **82**. Intramolecular cyclization would produce the corresponding isobenzofurane **83**, which alternatively, could also be formed by a concerted pathway. Then the fluoride induced ring opening and subsequent cyclization of intermediate **84** would generate phthalimide **79**.

Although most multicomponent reactions leading to isoindolinones make use of benzoic acid derivatives as one of the starting components, in a few contributions aromatic aldehyde, imine or nitrile compounds have been used instead.

**Scheme 25:**
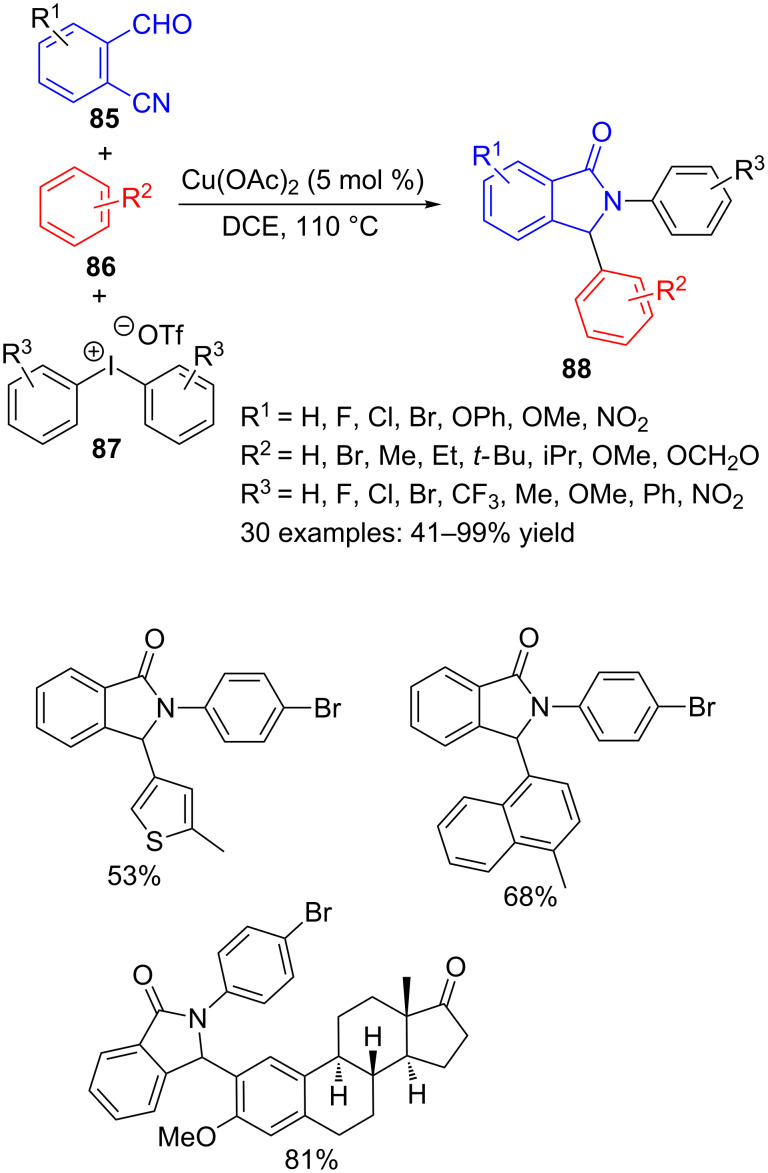
Copper-catalysed three-component reaction of 2-formylbenzonitriles **85**, arenes **86** and diaryliodonium salts **87**.

For instance, 2-formylbenzonitriles **85** along with a variety of arenes **86** and diaryliodonium salts **87**, combined in a copper-catalysed three-component cyclization produce 2,3-diarylisoindolinones **88** [103] (Scheme 25).

The scope of the reaction is very wide, since the three components have demonstrated the ability to be efficient sources of diversity in this reaction. Indeed, 33 isoindolinones **88** were prepared with yields ranging from moderate to very good. Even when unsymmetrical diaryliodonium salts **87** were used, the reaction was chemoselective, furnishing in good yields the products resulting from the transfer of the less hindered arene. However, this methodology exhibits two main limitations. First, arenes **86** with electron-withdrawing groups did not react under the optimized conditions and on the other hand, the atom economy is quite low, since a half of the diaryliodonium salt **87** is lost in the process.

**Scheme 26:**
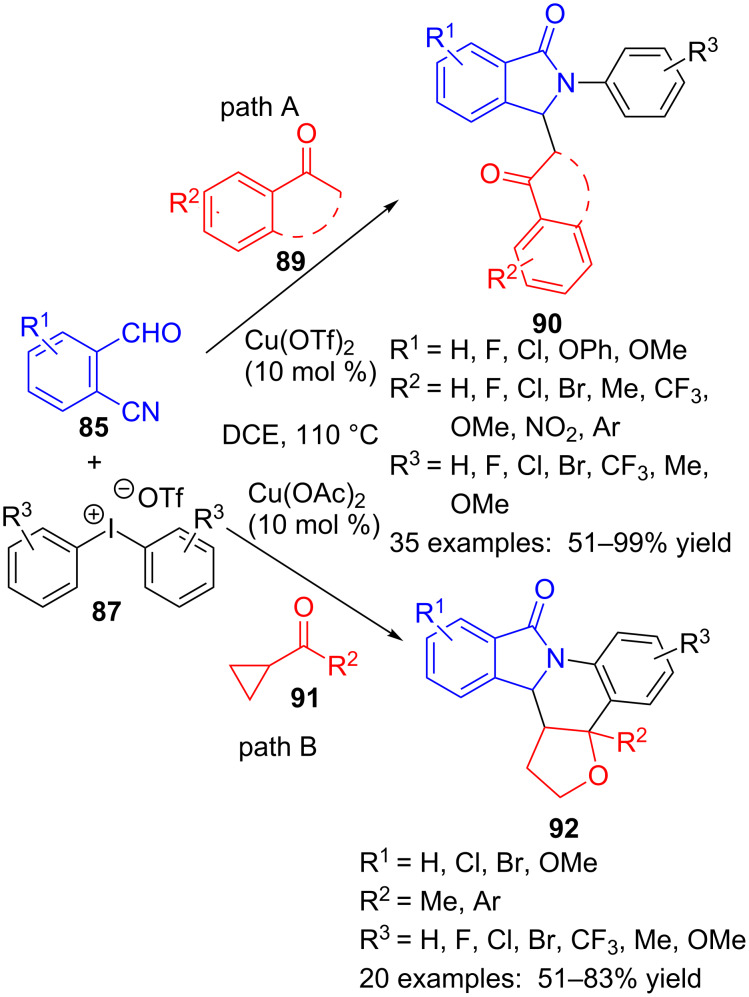
Copper-catalysed three-component reaction of 2-formylbenzonitriles **85**, diaryliodonium salts **87** and ketones **89** and **91**.

Replacing the arene substrate **86** by ketones, and keeping nearly equal reaction conditions, the same authors have achieved an efficient synthesis of 3-(2-oxopropyl)- or pentacyclic isoindolinones **90** or **92** (Scheme 26). Starting from several aryl and aliphatic ketones **89**, more than thirty isoindolinones **90** were obtained, with three points of diversity around the lactam core. Overall, aryl ketones containing electron-donating and electron-withdrawing functions and even a heteroaryl group delivered better yields than dialkyl ones (Scheme 26, path A) [104]. When cyclopropyl ketones **91** were used as substrates, a ring expansion and a new quaternary centre formation happened through the multicomponent reaction to produce pentacyclic derivatives **92** (Scheme 26, path B) [105].

**Scheme 27:**
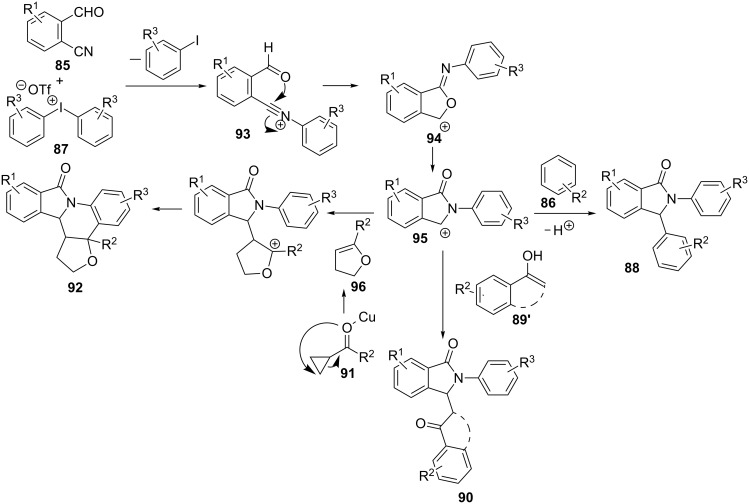
Proposed mechanism for the formation of 2,3-diarylisoindolinones **88**, **89** and **92**.

These reactions, either with arenes **86** or ketones **89** and **91**, seem to happen through an *N*-aryl nitrilium cation intermediate **93**, resulting from the reaction between formylbenzonitrile **85** and diaryliodonium salt **87** (Scheme 27). Intramolecular nucleophilic attack of the carbonyl group onto the nitrilium species would furnish cyclic intermediate **94**, that rearranges to afford cationic isoindolinone moiety **95**. Then, benzene derivative **86** would react by a Friedel–Crafts type reaction to form diaryl γ-lactam **88**. When ketone **89** is used instead of arene **86**, the enol form would act as nucleophile and upon reaction with carbocation **95**, compounds **90** could be isolated. Finally, cyclopropyl ketone **91** would first rearrange by copper catalysis and the so-obtained furane derivative **96** would add to the carbocation in **95**, followed by Friedel–Crafts cyclization, thus generating the polycyclic isoindolinones **92** in a formal hetero [4 + 2] cycloaddition process.

**Scheme 28:**
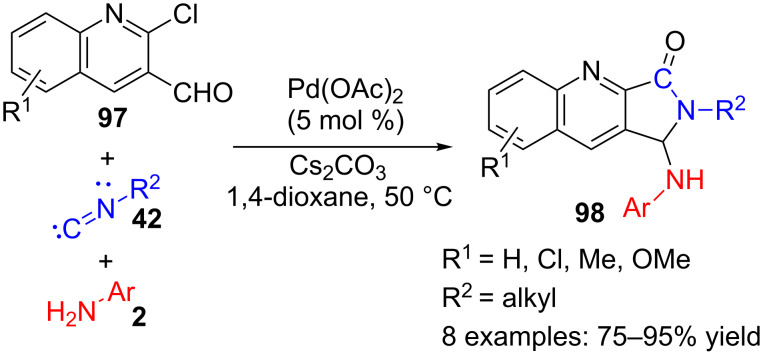
Palladium-catalysed three-component reaction of chloroquinolinecarbaldehydes **97** with isocyanides **42** and aromatic amines **2**.

Another aromatic aldehyde, in this case derived from a quinoline, can also be used as substrate for a multicomponent reaction, appropriate for the preparation of 3-aminoisoindolinone analogues **98** (Scheme 28) [106]. Indeed, the reaction of chloroquinolinecarbaldehydes **97** with isocyanides **42** and aromatic amines **2**, catalysed by Pd, produced a small collection (8 examples, 75–95% yield) of quinoline derivatives **98** containing a γ-lactam moiety with different substituents at the nitrogen of the 5-membered ring and in the γ-position. Although the authors do not suggest a mechanism, this probably starts with the formation of an imine by reaction of aromatic amine **2** with quinolinecarbaldehyde **97**.

**Scheme 29:**
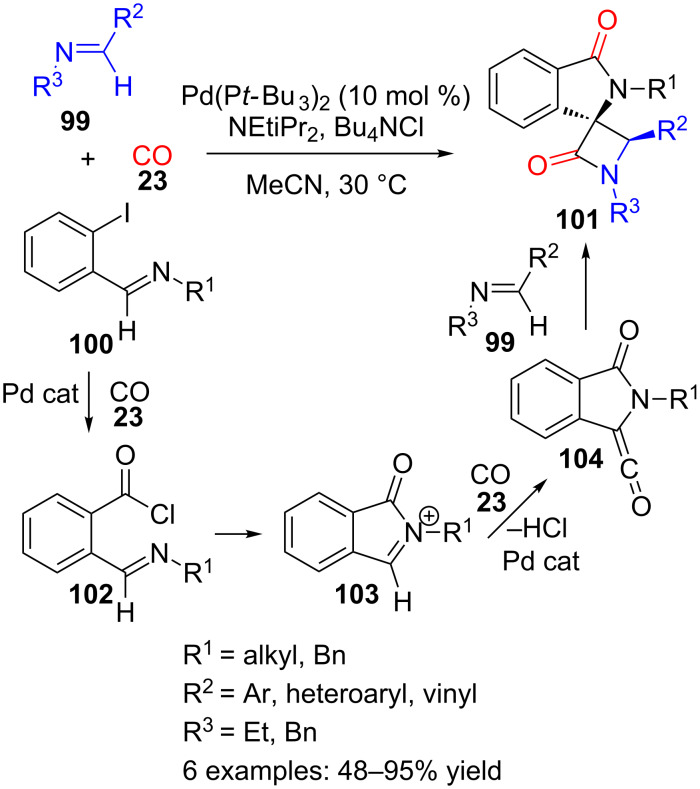
Palladium-catalysed three-component reaction of imines **99** with CO (**23**) and *ortho*-iodoarylimines **100**.

Certainly this is a quite feasible hypothesis, since it has been reported that *ortho*-iodo-substituted arylimines **100**, analogues of quinolinecarbaldehyde **97**, also reacted, under Pd catalysis, with CO (**23**) and imines **99** to furnish complex spirocyclic γ-lactams **101** (Scheme 29) [107]. These products show a *trans* orientation of the benzene moiety in the isoindolinone and the substituent in R^2^, according to NOE experiments and crystal structure analysis.

The proposed mechanism involves an initial palladium-catalysed carbonylation of iodoarylimine **100** to produce acid chloride **102** (Scheme 29). Intramolecular nucleophilic attack of the imine onto the acyl chloride would furnish cyclic *N*-acyliminium derivative **103**, which can then undergo a second palladium-catalysed carbonylation to form a stabilized ketene **104**. This is a good partner for a cycloaddition with an imine such as **99** that would give rise to the spiro β- and γ-lactam derivative **101** in a diastereoselective manner.

**Scheme 30:**
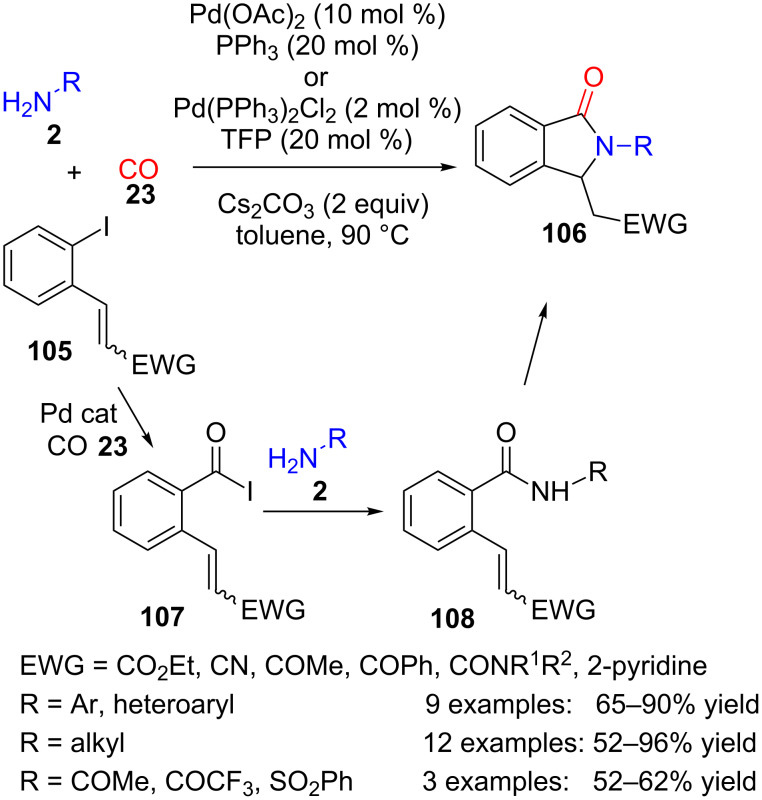
Palladium-catalysed three-component reaction of amines **2** with CO (**23**) and aryl iodide **105**.

Indeed, a seminal contribution also made use of a similar Pd-catalysed carbonylation followed by amide formation and cyclization in a three-component reaction between aryl iodides, incorporating a Michael acceptor **105**, amines and amides **2** and carbon monoxide (**23**) (Scheme 30) [108].

It is remarkable that not only aromatic but also aliphatic amines and even amides and sulfonamides can be used as the nitrogen-containing substrate. With chiral amines, very low diastereoselectivity was obtained, probably due to the harsh reaction conditions employed. In addition to carbonyl and carboxyl derivatives, pyridine was also used successfully as an electron-withdrawing group (EWG) in the Michael acceptor **105**. A simple control experiment allowed the authors to propose that the first stage of the reaction would be the insertion of carbon monoxide into the Ar–I bond to produce aryl iodide **107**, followed by the reaction with the nitrogen nucleophile to form amide intermediate **108**. Finally, intramolecular Michael addition would furnish lactam unit **106**.

### Oxindoles

**Scheme 31:**
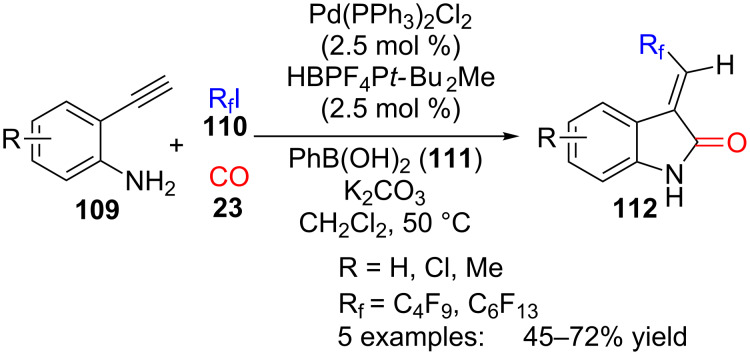
Three-component reaction of 2-ethynylanilines **109**, perfluoroalkyl iodides **110** and carbon monoxide (**23**).

The simplest protocol for the multicomponent assembly of oxindole heterocycles is the palladium-catalysed reaction involving carbon monoxide, in addition to terminal alkynes, arylboronic acids and alkyl iodides, which has been applied to the preparation of fluorinated 3-methyleneoxindole derivatives (Scheme 31) [109]. In this three-component reaction, 2-ethynylanilines **109** reacted with carbon monoxide (**23**) and perfluoroalkyl iodides **110**, in the presence of Pd(II) and phenylboronic acid (**111**), by means of an intramolecular amino-carbonylation reaction. Although phenylboronic acid did not incorporate into the final product structure, it was necessary for the reaction to take place. Using this protocol five oxindole derivatives **112** were synthesized with moderate yields, including one substrate containing a ^13^C-labelled carbon, suitable to be used as a metabolic tracer.

Nevertheless, most of the known multicomponent methods for the preparation of 2-oxindoles are based on the use of *N*-aryl amides as the main partner of the reagent pool.

**Scheme 32:**
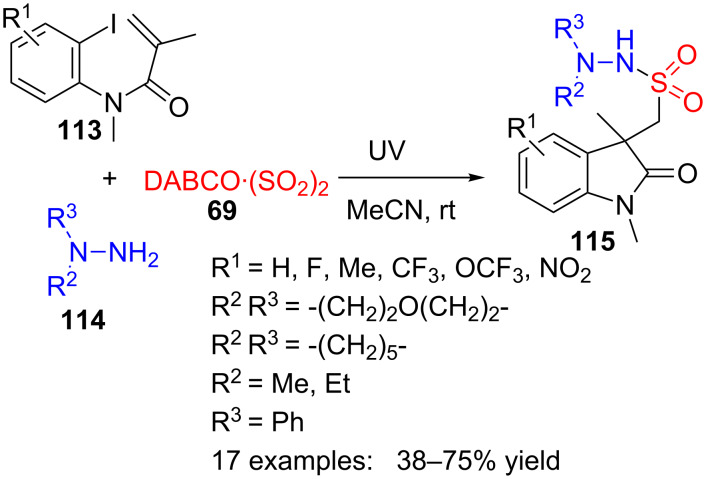
Ultraviolet-induced three-component reaction of *N*-(2-iodoaryl)acrylamides **113**, DABCO·(SO_2_)_2_ (**69**) and hydrazines **114**.

Wu et al. reacted *N*-(2-iodoaryl)acrylamides **113**, DABCO·(SO_2_)_2_ (**69**, also known as DABSO) as a surrogate of sulfur dioxide and hydrazine **114** in a photoinduced, catalyst-free three-component reaction (Scheme 32) [110]. In this way, a variety of (2-oxoindolin-3-yl)methanesulfonohydrazides **115** with diverse substituents in the aromatic ring and hydrazine nitrogen, were prepared with moderate to good yields.

**Scheme 33:**
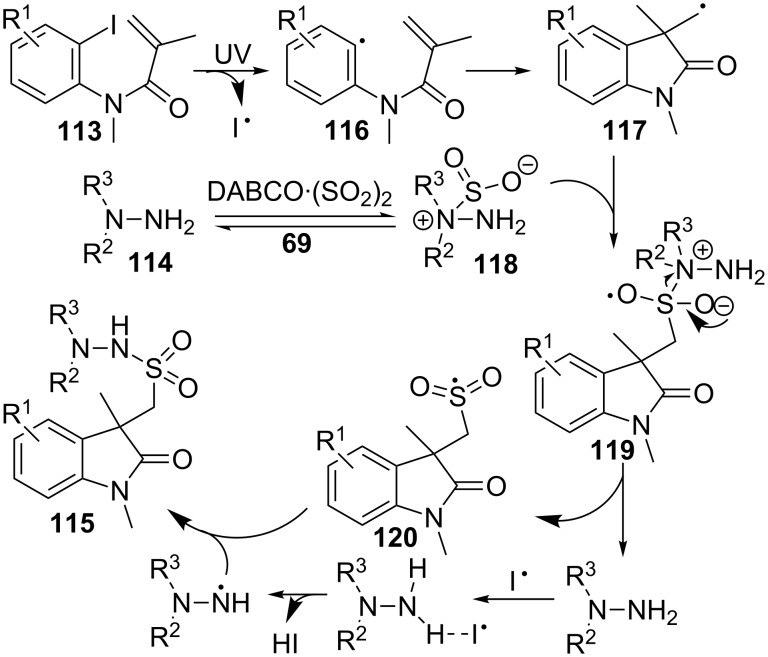
Proposed mechanism for the preparation of oxindoles **115**.

This transformation may be explained by a radical process promoted by UV irradiation, with an initial formation of aryl radical **116** from the corresponding aryl iodide **113** (Scheme 33). This radical would cyclize in an intramolecular 5-*exo* mode to furnish cyclic radical **117** which, in turn, can be caught by intermediate **118**, formed by hydrazine **114** and sulfur dioxide (Scheme 33). Rearrangement of the so-obtained intermediate **119**, through radical **120**, would provide oxindole **115**.

**Scheme 34:**
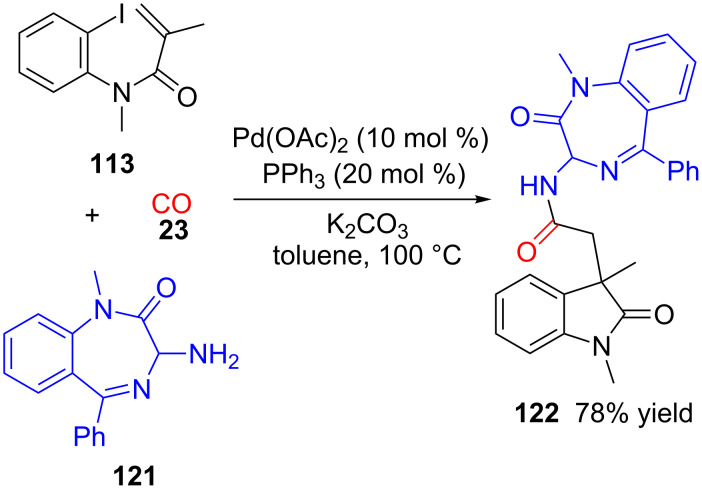
Three-component reaction of acrylamide **113**, CO (**23**) and 1,4-benzodiazepine **121**.

The same acrylamide **113** (R^1^ = H) has been recently used in another multicomponent synthesis along with CO (**23**) and benzodiazepine derivative **121** under palladium catalysis to give a 1:1 mixture of diastereoisomers of oxindole **122** with good yield (Scheme 34) [111]. In this case, the process consists in a palladium-catalysed cyclization followed by a carbonylation and anion capture.

**Scheme 35:**
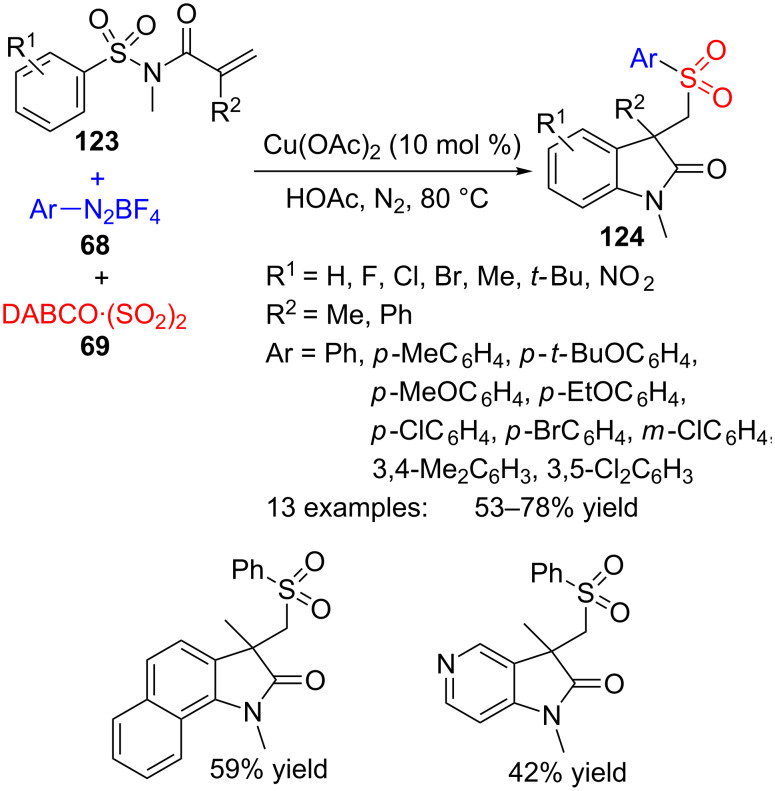
Multicomponent reaction of sulfonylacrylamides **123**, aryldiazonium tetrafluoroborates **68** and DABCO·(SO_2_)_2_ (**69**).

Several analogues of (2-oxoindolin-3-yl)methanesulfonohydrazides **115** have been prepared by another type of three-component approach, in this case under copper catalysis (Scheme 35) [112]. The partners of the reaction are *N*-(arylsulfonyl)acrylamides **123**, aryldiazonium tetrafluoroborates **68** and DABCO·(SO_2_)_2_ (**69**), as a source of sulfur dioxide. In this way, sulfonated oxindoles **124** are prepared in moderate to good yields.

A wide scope of aryldiazonium reagents **68** bearing electron-donating and electron-withdrawing groups worked well in the reaction, but when a pyridyl heterocycle was employed, the reaction failed. On the acrylamide side, activating and deactivating groups worked similarly well and even a *N*-heteroaryl analogue of **124** was obtained, although the yield was moderate (42%).

**Scheme 36:**
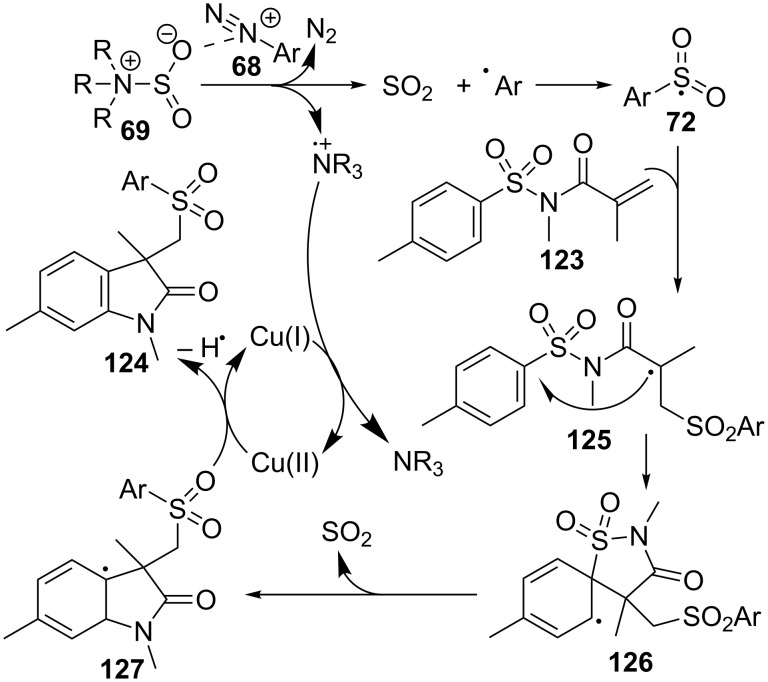
Proposed mechanism for the preparation of oxindoles **124**.

The formation of compound **124** may be explained by a radical process, starting with the addition of arylsulfonyl radical **72**, formed from **69** and aryldiazonium cation **68**, onto the alkene moiety of sulfonylacrylamide **123** (Scheme 36). Then, ipso-cyclization of radical **125** to **126**, departure of SO_2_ and final oxidation of **127** by Cu(II) would provide oxindole **124**. As a consequence of this desulfonylative 1,4-aryl migration, the SO_2_ group initially present in the starting acrylamide is replaced by another SO_2_ moiety coming from the DABCO·(SO_2_)_2_ reagent.

**Scheme 37:**
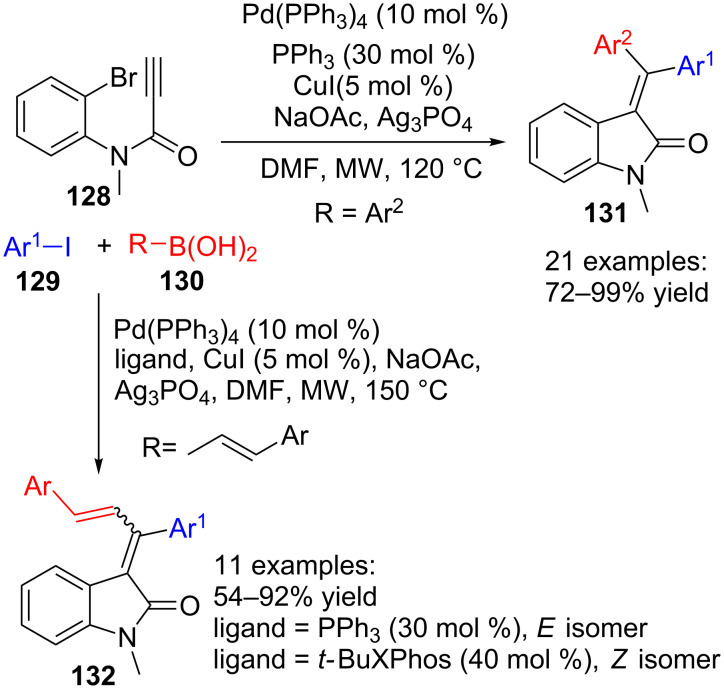
Three-component reaction of *N*-arylpropiolamides **128**, aryl iodides **129** and boronic acids **130**.

Other analogues of acrylamides that are appropriate substrates for oxindole preparation using multicomponent protocols are *N*-arylpropiolamides. This approach has been reported by Seo and co-workers in a series of papers that develop a three-component transformation comprising three palladium-catalysed reactions: Sonogashira, Heck and Suzuki–Miyaura (Scheme 37) [113–115]. In this methodology, *N*-arylpropiolamides **128** reacted with aryl iodides **129** and aryl- or styrylboronic acids **130** under microwave activation to yield 3-(diarylmethylene)oxindoles **131** or 3-(1,3-diarylallylidene)oxindoles **132**, respectively.

Initially [114–115], using arylboronic acids **130** (R = Ar^2^), a variety of twenty-one diarylmethylene oxindoles **131** were obtained with good yields. When aryl iodide and arylboronic acids bearing different substituents are used, the expected stereochemistry of the asymmetric products **131** is mainly obtained, where the aromatic group coming from the boronic acid partner settles far from the carbonyl group. Methoxy, nitro, chloro and acetoxy groups of the aromatic moiety can be located at the *ortho*, *meta* and *para* positions, although 2-nitro derivatives gave low yields or no reaction in some cases. In addition to above mentioned benzene derivatives, heteroaromatic boronic acids also worked well under the reaction conditions and provided high yields of the corresponding oxindoles **131**. Finally, switching the *N*-substituent in the starting propiolamide **128** by a H or a Bn group, did not affect the yield of the reaction, although the stereoselectivities diminished.

Next [113], the authors applied the reaction conditions to the use of styrylboronic acids **130** (R = CH=CHAr), and a collection of eleven 3-(1,3-diarylallylidene)oxindoles **132** were obtained with good yields and controlled stereochemistry (Scheme 37). Indeed, they found that the PPh_3_ ligand promoted the formation of the *E*-isomer as the main compound while the *t*-BuXPhos ligand induced the preferential formation of the *Z-*isomer.

**Scheme 38:**
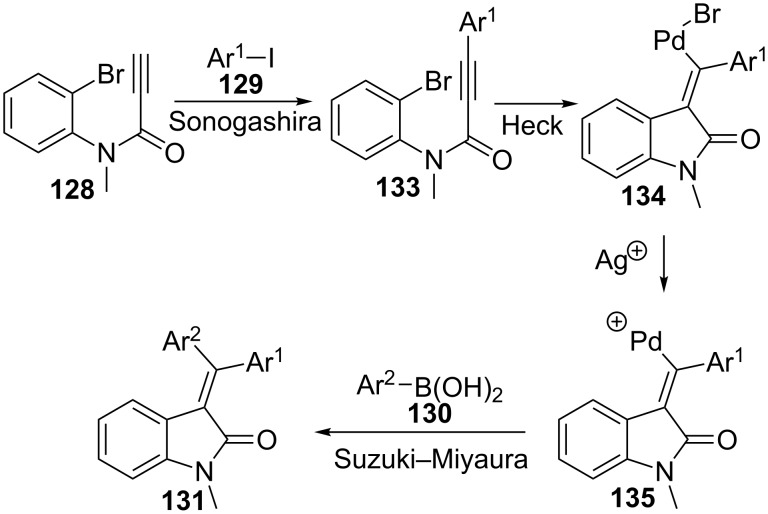
Proposed mechanism for the formation of diarylmethylene- and diarylallylideneoxindoles **131** and **132**.

As already mentioned before, this transformation is the result of a sequence of three palladium-catalysed reactions (Scheme 38). The first one is a Sonogashira coupling reaction between the terminal alkyne of propiolamide **128** and aryl iodide **129**, which is preferred to the Suzuki–Miyaura reaction between aryl iodide **129** and boronic acid **130** present in the reaction mixture. Then, an internal Heck cyclization reaction between the substituted alkyne and aryl bromide in **133** takes place to form a cyclic palladium intermediate **134** with *E*-configuration, resulting from a *syn*-addition mechanism of this step. The addition of a silver salt reduces the probability of isomerization of the double bond, presumably by changing the anionic character of the palladium complex to cationic in intermediate **135**. Finally, the Suzuki–Miyaura coupling of palladium salt **135** with boronic acid derivative **130** would provide the final oxindoles **131** or **132**.

**Scheme 39:**
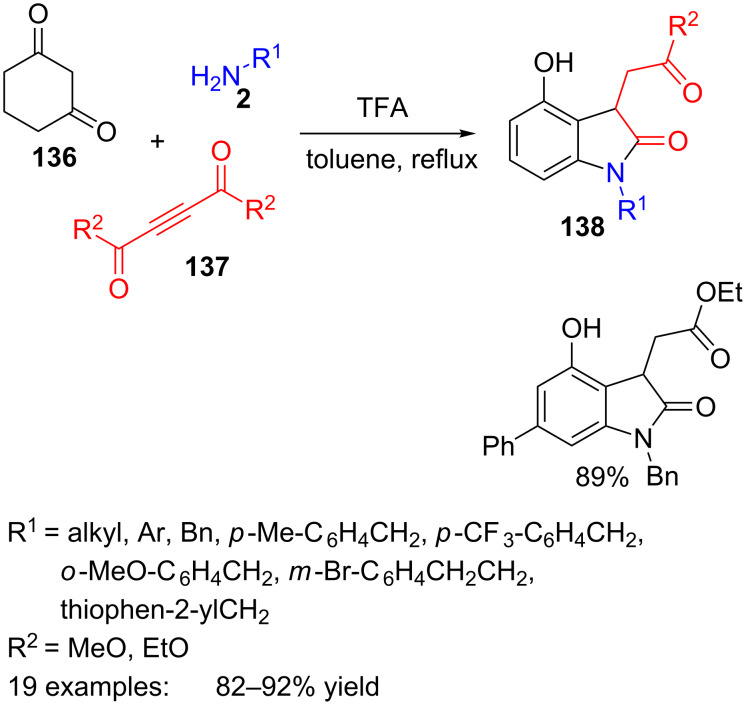
Three-component reaction of cyclohexa-1,3-dione (**136**), amines **2** and alkyl acetylenedicarboxylates **137**.

Finally, is worth mentioning an example of a multicomponent synthesis where the benzene moiety is created from non-aromatic substrates. Certainly, this three-component protocol includes an aromatization step in the course of the acid-catalysed reaction of cyclohexa-1,3-dione (**136**), amines **2** and alkyl acetylenedicarboxylates **137**, to produce the final phenolic oxindoles **138** with good yields (Scheme 39) [116].

Up to twenty different oxindole derivatives were prepared with any kind of substituents on the nitrogen atom, including aromatic, benzyl, heteroarylmethyl and alkyl groups.

**Scheme 40:**
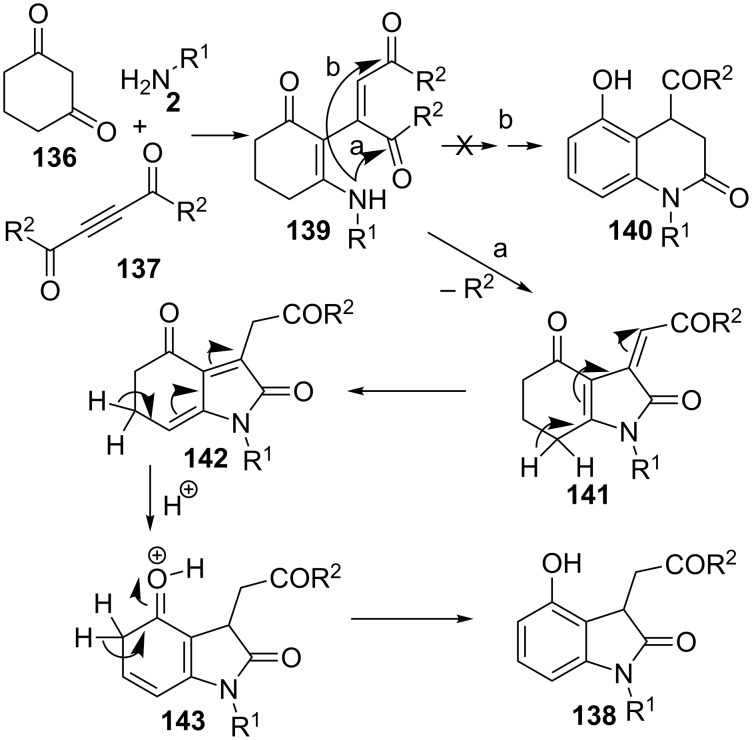
Proposed mechanism for the formation of 2-oxindoles **138**.

According to some control experiments, the authors propose a plausible mechanism involving a sequential enamine formation-Michael addition to produce intermediate **139**, followed by intramolecular cyclization to **141** and aromatization through species **142** and **143** (Scheme 40). The cyclization step takes place in a regioselective manner, leading to five-membered heterocycle **141** rather than to the formation of six-membered lactam **140**. Then, tautomerization followed by aromatization would provide oxindole **138**.

## Conclusion

Although the utilization of multicomponent reactions in synthesis is not a new deal, in the last years there has been an increased use of this strategy, particularly for the preparation of heterocyclic compounds. This is mainly due to the need to find new efficient methods in order to save raw materials and work time. Without doubt, multicomponent approaches in chemical synthesis take the advantage of those two saving features. This economical profit is especially interesting for the large-scale synthesis in pharmaceutical laboratories and industry.

Nevertheless, among the multicomponent synthetic methods available for the preparation of isoindolinones **II** and 2-oxindoles **III**, only one is enantioselective, even though many of the reactions described in this review involve the generation of new chiral centres. This drawback needs to be addressed so that new ligands and organocatalysts can be discovered and applied to the synthesis of enantiomerically pure γ-lactams of this type under smooth and environmentally more benign reaction conditions.

Another area of improvement is the need to more methods for the multicomponent building of the oxindole core, since the few approaches now available depart from complex starting materials. Therefore, it would be highly desirable to develop new protocols in order to increase the structural diversity of oxindole derivatives to be made using simple substrates and reagents.
